# Structural Characterization and Ameliorative Effects of *Mesona chinensis* Benth Polysaccharide Against Deoxynivalenol-Induced Oxidative Stress in Intestinal Epithelial Cells

**DOI:** 10.3390/nu17162592

**Published:** 2025-08-09

**Authors:** Ai-Hua Zhong, Qiu-Yun Li, Hua Su, Li-Jun Huang, Quan Zhou, Xiao-Dan Wang, Jia Song, Yong-Ning Wu, Xing-Fen Yang, Wei-Liang Wu

**Affiliations:** 1 Food Safety and Health Research Center, NMPA Key Laboratory for Safety Evaluation of Cosmetics, Guangdong-Hongkong-Macao Joint Laboratory for Contaminants Exposure and Health, Guangdong Provincial Key Laboratory of Tropical Disease Research, School of Public Health, Southern Medical University, Guangzhou 510515, China; zah_1095355051@163.com (A.-H.Z.); leeqy1218@163.com (Q.-Y.L.); yangalice79@smu.edu.cn (X.-F.Y.); 2 Public Health Service Center, Bao’an District, Shenzhen 518126, China; 3 Guangzhou Center for Food and Drug Evaluation, Guangzhou 510642, China; 4 NHC Key Laboratory of Food Safety Risk Assessment, Chinese Academy of Medical Science (2019RU014), China National Center for Food Safety Risk Assessment, Beijing 100022, China

**Keywords:** *Mesona chinensis* Benth polysaccharide, structure identification, deoxynivalenol, oxidative stress, intestinal epithelial cell

## Abstract

**Objectives**: Deoxynivalenol (DON) is a ubiquitous mycotoxin detected in the environment and foodstuffs. DON exposure can lead to chronic intestinal inflammation. Therefore, intervention strategy needs to be established to prevent the intestinal inflammation caused by DON. **Methods**: The structure of *Mesona chinensis* Benth polysaccharide-3 (MCP-3), a major component isolated and purified from crude MCP, was analyzed using spectroscopic and chromatographic methods. In vitro assays were conducted on the potential antioxidant bioactivities of MCP-3 and its ameliorative effects on deoxynivalenol-induced oxidative stress in intestinal epithelial cells. **Results**: Saline-eluted MCP-3 was identified as an acidic heterogeneous polysaccharide with an average molecular weight of 16.014 kDa. Its major monosaccharide components were glucose (20.19%), galactose (11.82%), rhamnose (17.23%), galacturonic acid (29.72%), arabinose (7.11%), xylose (8.09%), mannose (2.79%), and glucuronic acid (3.04%). The main backbone of MCP-3 was composed of the following sequence: →4)-α-D-GalpA-6-(1→4)-α-GalpA-(1→4)-α-D-GalpA-6-(1→2)-α-L-Rhap-(1→4)-α-D-GalpA-6-(1→2,4)-α-L-Rhap-(1→. MCP-3 showed strong antioxidant ability in in vitro assays. It effectively prevented redox imbalance induced by the mycotoxin deoxynivalenol in intestinal epithelial cell models based on Caco-2 and NCM460 cells. MCP-3 significantly increased (*p* < 0.05) the activities of superoxide dismutase, glutathione peroxidase, and catalase, and significantly decreased (*p* < 0.05) the levels of malondialdehyde and reactive oxygen species, thereby improving redox homeostasis. **Conclusions**: MCP-3 has potential as a natural antioxidant for use in functional food and nutraceutical industries to help regulate intestinal oxidative stress caused by mycotoxin DON.

## 1. Introduction

Intestine shoulders great responsibilities for digestion and immunity [1]. The intestinal epithelium acts as a selective barrier, allowing nutrient transport through enterocytes while preventing toxic xenobiotics, pathogenic bacteria, and their associated toxins from entering the circulatory system [2]. However, when these harmful substances directly interact with enterocytes in the gastrointestinal tract, they disrupt gut homeostasis, resulting in chronic intestinal inflammation, which imposes a substantial global disease burden [3,4]. Recent extensive literature has highlighted that various contaminants in foods, such as heavy metals, mycotoxins, persistent organic pollutants, and agrochemical residues, are substantial pathogenic factors posing serious threats to gut health and have attracted considerable attention [5,6,7,8,9,10,11].

Deoxynivalenol (DON), also known as vomitoxin, is a fungal secondary metabolite mainly produced by *Fusarium graminearum* and *Fusarium culmorum* [12,13]. It is the most prevalent trichothecene mycotoxin contaminating cereal crops, such as wheat, corn, barley, and oats. High-exposure regions to DON have been identified globally, with developing countries being particularly affected [14,15]. It is comprehensively documented that chronic DON exposure via diet can cause a wide range of deleterious effects in humans, including immunotoxicity and enterotoxicity [11,13,16,17,18]. Upon dietary exposure, DON passes through the digestive tract along with the contaminated food, and after being released from the food matrix via digestion, it first targets the intestine, the largest immune organ in the body. The dissociated DON disrupts intestinal homeostasis by triggering an apoptotic cascade in intestinal epithelial cells, leading to damage of intestinal integrity, disruption of tight junctions, and increased intestinal permeability. These changes ultimately lead to chronic intestinal inflammation and even severe diseases such as inflammatory bowel disease and colorectal cancer [6,11,18,19]. The underlying mechanism of deoxynivalenol-induced immunotoxicity is widely attributed to excessive reactive oxygen species (ROS) generated by deoxynivalenol-induced oxidative stress, which significantly contributes to an inappropriate and sustained immune response in the intestinal epithelium [3,7,16]. Therefore, effectively scavenging ROS and regulating inflammation is believed to be a crucial strategy for protecting against intestinal injury caused by chronic low-dose dietary DON exposure.

*Mesona chinensis* Benth (*M. chinensis* Benth), a traditional local herb commonly used in soups and pastries in Southern and Southeastern China, has been officially recognized as a food and medicine homology resource. Traditionally, it has been used to alleviate sore throat, quench thirst and heat, and detoxify heat-related knot toxins [20], owing to its anti-inflammation effects exerted by diverse bioactive ingredients that offer potential health-promoting benefits, such as polysaccharides [21,22,23,24,25,26], flavonoids [27], phenols [28], and other compounds. However, it is still necessary to fully explore the component exhibiting antioxidant and anti-inflammatory bioactivities in *M. chinensis* Benth. As the major component, polysaccharides extracted from *M. chinensis* Benth may have potential ameliorative effects on oxidative stress according to the previous in vitro and in vivo studies [20,21,22,23,24,25,26,27,28,29,30]. However, to our knowledge, the potential ameliorative effects of *M. chinensis* Benth polysaccharide (MCP) on oxidative stress in intestinal epithelial cells—particularly in preventing deoxynivalenol-induced intestinal inflammation—still need to be elucidated further. Moreover, relevant research remains limited, particularly regarding the structure-function relationship involved in the modulation of intestinal oxidative stress.

Accordingly, one of the objectives of this study was to elucidate the structural characteristics of MCP-3 eluted from diethylaminomethyl cellulose-52 (DEAE-52) and further purified by Dextran 100 gel permeation chromatography. In addition, the in vitro antioxidant bioactivities of MCP-3 were evaluated using various verification methods. Furthermore, the ameliorative effects of MCP-3 on DON-induced oxidative stress in intestinal epithelial cells were illuminated to explore the relationship between polysaccharides and intestinal health. This will aid in the establishment of intervention strategies for individuals with chronic intestinal inflammation caused by DON.

## 2. Materials and Methods

### 2.1. Materials and Reagents

The *M. chinensis* Benth used in this study, which was planted at Dengluxia Village, Paitan, Zengcheng District, Guangzhou City, and harvested in October 2020, was supplied by Guangzhou Nature Family Farm Limited (Guangzhou, China). DON (purity, ≥98%) was purchased from Sigma-Aldrich (St. Louis, MO, USA). Monosaccharide standards and dialysis bags (MW > 3000 Da) were purchased from Yuanye Bio-Technology Co., Ltd. (Shanghai, China). DEAE-52 and Dextran 100 were obtained from Aisha Biotechnology Co., Ltd. (Guangzhou, China). Assay kits for malondialdehyde (MDA), glutathione peroxidase (GSH-Px), catalase (CAT), superoxide dismutase (SOD), and ROS were acquired from Multisciences (Lianke) Biotech, Co., Ltd. (Hangzhou, China). 2, 5-diphenyltetrazolium (MTT) for cytotoxicity was provided by Sigma-Aldrich (St. Louis, MO, USA). Ascorbic acid and butylated hydroxytoluene (BHT) were obtained from Solexpo Technology Co. (Beijing, China). Eagle’s Minimum Essential Medium (EMEM), Dulbecco’s Modified Eagle’s Medium (DMEM), and fetal bovine serum (FBS) were obtained from Gibco Life Technologies (Waltham, MA, USA). The bicinchoninic acid (BCA) protein concentration assay kit was purchased from Beyotime Biotechnology Co., Ltd. (Shanghai, China), whereas the cDNA Reverse Transcription Kit, SYBR Green Pro Taq HS Premix qPCR Kit were obtained from Accurate Biology Engineering Co., Ltd. (Changsha, China). The ROS fluorescent probe dichlorodihydrofluorescein diacetate (DCFH-DA) was synthesized by Landyi Biotechnology Co., Ltd. (Suzhou, China). All the other chemicals and reagents used for the structural characterization of MCP and the evaluation of its in vitro antioxidant activities were of analytical, biochemical, or chromatographic grade, purchased from qualified suppliers.

### 2.2. Extraction Procedure of MCP

The whole dried *M. chinensis* Benth plant was roughly ground and passed through a 60-mesh sieve to collect *M. chinensis* Benth powder for polysaccharide extraction. The extraction process was performed using the method described by Huang et al., with minor modifications [24]. The resulting crude MCP was harvested through alkali extraction followed by alcohol precipitation. The precipitates were redissolved into ultrapure water (<0.057 μS/cm) for deproteinization, dialysis, and lyophilization.

### 2.3. Separation and Purification of MCP

The polysaccharide fraction was separated from crude MCP using a DEAE-52 purification column with a loading volume of 200 mg, and eluted sequentially with pure water, and 0.1, 0.2, and 0.4 mol/L NaCl solutions at a flow rate of 1 mL/min, according to the method reported by Zhang et al. [31] and Liu et al. [32]. The elution curve was plotted with tube number on the horizontal axis and absorbance measured using the phenol sulfuric acid method on the vertical axis. A fraction corresponding to a single elution peak was collected for dialysis desalting, rotary concentration, and lyophilization. Each fraction was further purified using a Dextran 100 column.

### 2.4. Physicochemical Determination of Polysaccharide

#### 2.4.1. Physicochemical Determination

The polysaccharide yield was measured using the phenol–sulfuric acid method according to Huang et al. [24]. Briefly, the absorbances of varying polysaccharide concentrations were measured at 490 nm after reaction with 6% (*m*/*v*) phenol aqueous solution and concentrated sulfuric acid.

#### 2.4.2. Protein Content

The protein content in the polysaccharide was determined using the Coomassie Brilliant Blue G250 staining method. The absorbance was detected at 595 nm after mixing the polysaccharide sample with 0.2% (*m*/*v*) Coomassie Brilliant Blue G250 dye aqueous solution. The protein content in the polysaccharide was calculated based on a protein standard curve.

#### 2.4.3. Ultraviolet Absorption Spectrum

To confirm the removal of protein and nucleic acid residues in the polysaccharide, the absorbance of 0.1% (*m*/*v*) MCP-3 aqueous solutions was measured at a wavelength ranging from 200 to 400 nm using an ultraviolet-visible spectrophotometer (UV-1600, Shimadzu, Tokyo, Japan). An appropriate aliquot of each solution was added to a cuvette prior to measurement.

### 2.5. Structural Identification of MCP-3

#### 2.5.1. Congo Red Test

To confirm the stereochemical structure of the polysaccharide, the Congo Red test was performed following a previously reported method with minor modifications [23]. A 1.7-mL aliquot of the MCP-3 solution (1 mg/mL) was mixed with 200 µL of 60 µmol/L Congo Red solution. Then, 2 mol/L NaOH solution was added stepwise to adjust the final NaOH concentration to 0, 0.05, 0.1, 0.2, 0.3, 0.4, 0.5, and 0.6 mol/L. After the reaction was completed, the maximum absorption was scanned over the 190–1100 nm wavelength range using an ultraviolet-visible spectrophotometer (UV-1600).

#### 2.5.2. Infrared Spectroscopic Analysis

The Fourier transform infrared spectroscopy (FT-IR) spectrum of MCP-3 was recorded using a Nicolet iS10 FT-IR spectrometer (Thermo Nicolet Corporation, Waltham, MA, USA) at a scanning range of 4000–400 cm^−1^. The spectrometer had a resolution of 4 cm^−1^ and a signal-to-noise ratio (S/N) of 50,000:1. Before measurement, the sample was ground with potassium bromide powder and pressed into pellets using the potassium bromide disc technique.

#### 2.5.3. Molecular Weight Determination

The average molecular weight of the MCP-3 polysaccharide was analyzed using a Waters 1515 high-performance gel permeation chromatography system (HPGPC, Waters Corporation, Milford, MA, USA), equipped with three tandem columns (OHpak SB-803 HQ, OHpak SB-804 HQ, and OHpak SB-805 HQ, Shodex, Tokyo, Japan), a Waters 2410 differential refraction detector (Waters Corporation, Milford, MA, USA), and a Waters 2707 auto injector (Waters Corporation). A 30-μL aliquot of the 5 mg/mL sample was injected into the HPGPC system maintained at 40 °C. Separation was carried out using a 0.05 mol/L NaCl aqueous solution as the mobile phase at a flow rate of 0.65 mL/min. Calibration was performed using T-series dextran standards with molecular weights of 5, 12, 25, 50, 80, 150, 270, 410, and 670 kDa (Sigma-Aldrich, Darmstadt, Germany) dissolved in 0.05 mol/L NaCl aqueous solution.

#### 2.5.4. Determination of Monosaccharide Composition

The monosaccharide composition of MCP-3 was determined using a previously described method, with minor modifications [1]. Instrumental analysis was conducted using a Thermo U3000 liquid chromatography system (Thermo Fisher Scientific, Waltham, MA, USA) equipped with a ZORBAX Eclipse XDB C_18_ column (4.6 mm × 250 mm, 5.0 μm, Agilent Technologies, Inc., Santa Clara, CA, USA) maintained at 30 °C. A 10-μL aliquot of the sample solution was injected into the column for isocratic elution using a mobile phase comprising acetonitrile and 12 g/L of KH_2_PO_4_ buffer (pH 6.8) in a volume ratio of 17:83, at a flow rate of 0.8 mL/min. The eluted monosaccharide was detected at 250 nm.

#### 2.5.5. Methylation Analysis

Methylation analysis of MCP-3 was conducted according to a reported protocol [1]. The glycosidic linkages of MCP-3 were analyzed using an Agilent 7890-5997B (Agilent Technologies Inc., Santa Clara, CA, USA) gas chromatography–mass spectrometry system (GC-MS) equipped with a HP-5 MS column (30 m × 0.25 m × 0.25 μm) and an Agilent quadrupole mass detector. The temperature program was initiated at 50 °C for 1.0 min, ramped to 130 °C at a rate of 50 °C /min, then increased to 230 °C at 3 °C/min, and held for 2 min. The mass spectrometer operated in full scan mode, and with a mass-to-charge (*m*/*z*) range of 30–600.

#### 2.5.6. Nuclear Magnetic Resonance Spectroscopy

The lyophilized MCP-3 sample was dissolved in 0.5 mL of D_2_O, and 3-(trimethylsilyl) propionic acid-d4 sodium salt (TMSP) was added as an internal standard for nuclear magnetic resonance (NMR) analysis at 70 °C. The ^1^H-NMR, ^13^C-NMR, and distortionless enhancement by polarization transfer 135 (DEPT-135) of NMR-1D spectra, correlated spectroscopy (^1^H–^1^H COSY), nuclear overhauser effect spectroscopy (NOESY), heteronuclear single quantum coherence spectroscopy (HSQC), and heteronuclear multiple bond correlation spectroscopy (HMBC) of NMR-2D spectra were registered on a Bruker AVANCE HD III 600 MHz NMR Spectrometer (Bruker, Berlin, Germany) [23]. The calibrations were as follows: HDO hydrogen δ H = δ 4.70 ppm, methyl carbon δ C = δ − 1.80 ppm for TMSP.

#### 2.5.7. Scanning Electron Microscope

An appropriate quantity of completely lyophilized polysaccharide was positioned on a copper sheet for secure attachment and fixation, and then the sample was uniformly coated with a thin gold layer in a gold-plating chamber using a scanning electron microscope (SEM) gold-spraying device (MC1000, Hitachi Ltd., Tokyo, Japan). Finally, images of the polysaccharide morphological features were viewed with a magnification of 100× and 400× at an accelerating voltage of 15.0 kV using a scanning electron microscope (S-3000N, Hitachi Ltd., Tokyo, Japan).

### 2.6. In Vitro Antioxidant Ability of MCP-3

The in vitro antioxidant ability of MCP-3 was measured using a BioTek EPOCH 2 microplate reader (Agilent Technologies, Inc., Santa Clara, CA, USA). Ascorbic acid and BHT were selected as positive controls for comparison with the antioxidant ability of MCP-3.

#### 2.6.1. 2,2-Diphenyl-1-picrylhydrazyl (DPPH) Free-Radical Scavenging Ability

The DPPH radical scavenging ability of DPPH was measured using the protocol reported by Tang et al., with slight modifications [29]. The MCP-3 solution was fully mixed with a DPPH reagent. The mixture was measured at a wavelength of 517 nm after being left to stand for 60 min in the dark at room temperature.

#### 2.6.2. Superoxide Free-Radical Scavenging Ability

To measure the capacity of MCP-3 to scavenge superoxide free radicals, polysaccharide samples were first added to Tris-HCl (50 mmol/L, pH = 8.2) to allow for co-incubation with pyrogallic acid in a water bath at 25 °C. The absorbances of the resulting final products were measured at 299 nm after the reactions were terminated by an HCl aqueous solution [23].

#### 2.6.3. Hydroxyl Free-Radical Scavenging Ability

The hydroxyl radical scavenging ability was assessed using the methods described by Yang et al. [33] and Tang et al. [34]. Briefly, polysaccharide solutions were thoroughly mixed with FeSO_4_ aqueous solution and salicylate ethanol solution, followed by incubation with H_2_O_2_ aqueous solution at 37 °C. After completion of the reaction, the absorbance was immediately measured using a BioTek EPOCH 2 microplate spectrophotometer at 510 nm.

#### 2.6.4. Nitroso Ion Scavenging Ability

According to the Chinese standard GB 5009.33-2016 [35], the nitroso ion scavenging rate was determined using the naphthalene hydrochloride ethylenediamine method. In summary, MCP-3 solutions were completely mixed with NaNO_2_ aqueous solution and then incubated in a water bath at 37 °C for 30 min. Next, the mixture was left to stand still for 5 min after being mixed with 1 mL of 4-aminobenzenesulfonic acid. Subsequently, the mixture was mixed with naphthalene ethylenediamine hydrochloride and left to stand for 15 min. Finally, the absorbance of the resulting mixture was measured at a wavelength of 540 nm.

#### 2.6.5. 2,2′-Azinobis-(3-ethylbenzthiazoline-6-sulphonate) (ABTS) Free-Radical Scavenging Ability

The measurement of the ABTS free radical scavenging rate of MCP-3 was conducted using a previously reported method [34]. Briefly, the ABTS aqueous solution was thoroughly mixed with a potassium persulfate aqueous solution in equal volume. The mixture was left in the dark at 4 °C for 16 h to obtain a stable absorbance at 734 nm. Next, the radical solution was further diluted with deionized water until the initial absorbance value of 0.7 ± 0.02 was reached at 734 nm. To analyze MCP-3, 3.9 mL of diluted ABTS solution was added to 0.1 mL of polysaccharide and then shaken well. Absorbance was observed after 6 min at 734 nm. Ascorbic acid and BHT were used as positive controls.

#### 2.6.6. 2-Phenyl-4,4,5,5-tetramethylimidazoline-3-oxide-1-oxyl (PTIO) Free-Radical Scavenging Ability

Assay of PTIO free-radical scavenging ability was performed using a method reported by Li et al. [36] and Yang et al. [37]. Initially, the PTIO reagent was fully vortexed with the sample solutions. After incubation at an ambient temperature for 30 min, the absorbance of the resulting mixture was measured at 557 nm using a BioTek EPOCH 2 microplate reader.

#### 2.6.7. Ferric Reducing Ability

In vitro ferric reducing ability of MCP-3 was measured following the protocol described by Oyaizu et al. [38]. In brief, sample solutions were thoroughly blended with sodium phosphate buffer (pH = 6.6) and 2.5 mL of 1% (*m*/*v*) potassium ferricyanide aqueous solution, and then incubated at 50 °C for 20 min. Subsequently, trichloroacetic acid aqueous solution was immediately added. Subsequently, the supernatants of 2.5 mL were mixed with 2.5 mL of deionized water and FeCl_3_ aqueous solution after centrifugation at 3000 rpm for 10 min. The mixture was allowed to stand for 10 min, and the absorbance was measured at 700 nm.

### 2.7. Ameliorative Effects of MCP-3 on DON-Induced Oxidative Stress and Inflammation in Caco-2 and NCM460 Cells

#### 2.7.1. Cell Culture

The Caco-2 cell line culture, grown in a basal medium EMEM (the American Type Culture Collection, Manassas, VA, USA, ATCC, 30-2003) supplemented with 20% fetal bovine serum (FBS, Gibco 10099-141), and the NCM460 cell line, grown in a basal medium consisting of DMEM (Gibco C11995500BT) supplemented with 10% FBS (Viva Cell C04001-500), were supplied by ATCC. All cells were maintained in a humidified incubator at 37 °C with 5% CO_2_ and 95% air. When cell confluence reached 80–90%, sub-culturing was performed after digestion with phosphate-buffered saline (PBS) containing 1-mmol/L ethylenediaminetetraacetic acid and 0.25% trypsin. The cells in the logarithmic growth phase were used for subsequent assays.

#### 2.7.2. Cell Proliferation Analysis

Cell viabilities were evaluated via cytotoxicity assay using the MTT method [31]. A 100-µL aliquot of Caco-2 and NCM460 cells (0.5–1 × 10^5^ cells/mL) was cultured in 96-well plates for 24 h before being treated with MCP-3 (0, 3.91, 15.63, 62.5, 250, and 1000 µg/mL) or DON (Caco-2: 0, 1, 3, 6, 9, 12, 15, 18, 21, and 24 µg/mL; NCM460: 0, 0.064, 0.32, 1.6, 8, 40, 200, and 1000 µg/mL). The cells were further incubated at 37 °C for 24 h. After discarding the medium and rinsing twice with PBS, 100 µL of medium containing 10% MTT solution (5 mg/mL) was added to each well and incubated in the dark for an additional 4 h. Subsequently, the MTT-containing medium was carefully removed, and 100 µL of dimethyl sulfoxide was added to each well to dissolve the formazan crystals formed by viable cells. Finally, cell viability was determined by measuring the absorbance at 490 nm using a BioTek EPOCH 2 microplate reader, followed by the dissolution of MTT formazan crystals in six replicates, and untreated cells served as the controls.

(1) $$Cell\;viability\;rate\left(\%\right)=\frac{A_{test}-A_{blank}}{A_{control}-A_{blank}}\times 100\%$$

where *A_test_* is the absorbance of the test well (including cells, cell culture, MTT solution, and DON or MCP-3), *A_blank_* is the absorbance of the blank well (including cell culture and MTT solution), and *A_control_* is the absorbance of the control well (including cells, cell culture, and MTT solution).

#### 2.7.3. Oxidative Stress of Intestinal Epithelial Cells Induced by DON

Caco-2 and NCM460 cells were seeded into a six-well plate at a density of 5 × 10^4^ cells/mL. After 24 h of attachment at the bottom of the plate, subconfluence cells were treated with DON and MCP-3 in fresh EMEM or DMEM for 12 h. The experiment included a control group (EMEM or DMEM without DON and MCP-3), a model group (5 µg/mL DON), and three treatment groups (low level: 5-µg/mL DON and 25-µg/mL MCP-3; medium level: 5-µg/mL DON and 100-µg/mL MCP-3; high level: 5-µg/mL DON and 400-µg/mL MCP-3). Then, the medium containing DON and MCP-3 was discarded, and the cells were washed twice with PBS (pH 7.4) before further analysis.

#### 2.7.4. Determination of GSH-Px, CAT, SOD, and MDA

After treatment, the cells were collected through centrifugation at 1000 rpm for 10 min, and then cell breakage was implemented by an SFX 20 ultrasonic cell fragmentation apparatus (Branson Ultrasonics Corp., St. Louis, MO, USA) in an ice-water bath at 230 V after the cells were resuspended in PBS. Finally, the supernatant was collected via centrifugation at 12,000 rpm for the analysis of GSH-Px, CAT, SOD, and MDA using commercially available bio-kits (Jiancheng Bioengineering Institute, Nanjing, China). The protein concentration was determined using the BCA method via a bio-kit. The absorbance was measured using an EPOCH 2 microplate reader (Bio-Tek, Winooski, VT, USA).

#### 2.7.5. ROS Measurement

The ROS accumulation in Caco-2 and NCM460 cells after treatment was detected by staining the cells with ROS fluorescent probe DCFH-DA, which was previously reported by Jin et al., with slight modifications [39]. A six-well plate with cell climbing slices inside was used for ROS measurement. Briefly, the cells were seeded into the six-well plate at a density of 5 × 10^4^ cells/mL for incubation for 12 h. Then, the cells were treated for 12 h and washed in triplicate with PBS. Next, the cells were incubated with 10 μmol/L DCFH-DA for 30 min at 37 °C. Subsequently, the cells were collected and resuspended after being washed in triplicate with PBS. An aliquot of 100-μL resuspension solution was added into a 96-well plate for the detection of fluorescent intensity using a BioTek SYNERGY H1 fluorescence microplate reader (Agilent Technologies, Inc., Santa Clara, CA, USA), whereas the slide covered with cells were observed green fluorescence in dark and imaged using an inverted fluorescence microscope Eclipse 80i (Nikon Corporation, Tokyo, Japan). The relative content of ROS in the cells was calculated using protein content for calibration:

(2) $$Relative\;content\;of\;ROS=\frac{B_{n}}{B_{0}}\times 100\;mg\;protein$$

where B_0_ is the fluorescence intensity of the control group, and B_n_ is the fluorescence intensity of the treatment group.

#### 2.7.6. Quantitative Analysis of Cytokines

The total RNA was obtained from different treatments with a TRIzol reagent according to a previously described method [1]. Subsequently, reverse transcription of RNA was performed using a cDNA reverse transcription kit after the purity and concentration of the total RNA were determined using an ultra-micro spectrophotometer. Finally, cDNA was amplified and quantified for the target gene according to the SYBR^®^ Green method using a SYBR Green Pro Taq HS Premix Kit (Accurate Biology, Changsha, China) by a Roche LightCycler 96 fluorescence quantitative PCR system (Basel, Switzerland). The reaction program of real-time PCR for mRNA relative expression and the primer sequences used for the analysis are listed in Table S1 and Table S2, respectively. The mRNA expressions were calculated using the 2^−△△CT^ method with β-actin as the reference gene. The IL-1β, IL-6, and TNF-α levels in two cell lines were measured using a commercial enzyme-linked immunosorbent assay kit, following the manufacturer’s instructions.

### 2.8. Statistical Analysis

Statistical analysis was conducted using SPSS version 25.0 (IBM, Armonk, NY, USA) and plotted using GraphPad Prism 9.0 (GraphPad Software, Boston, MA, USA). For continuous variable data, statistical significance was determined via one-way analysis of variance, followed by Dunnett’s test and unpaired two-tailed Student’s *t*-test. All the determination results were expressed as average values ± standard deviations from at least triplicate measurements, and statistical significance was defined as *** *p* < 0.001, ** *p* < 0.01, and * *p* < 0.05.

## 3. Results and Discussion

### 3.1. Extraction, Purification, and Composition of MCP

MCP was successfully extracted from *M. chinensis* Benth. As shown in Table S3, after dialysis and lyophilization, the MCP yield was 12.60 ± 0.33%, which was consistent with that reported by Tang et al. [29], and relatively higher than those reported by Chen et al. [21], Lin et al. [40], and Niu et al. [41].

Using the phenol–sulfuric acid method, the total sugar content of MCP was measured at 35.75 ± 4.55% (Table S3), while the protein content was 8.54 ± 1.33%, indicating that MCP is a polysaccharide. The total sugar content aligns with previous studies, although the polyphenolic content was lower [29,40,41].

**Figure 1:**
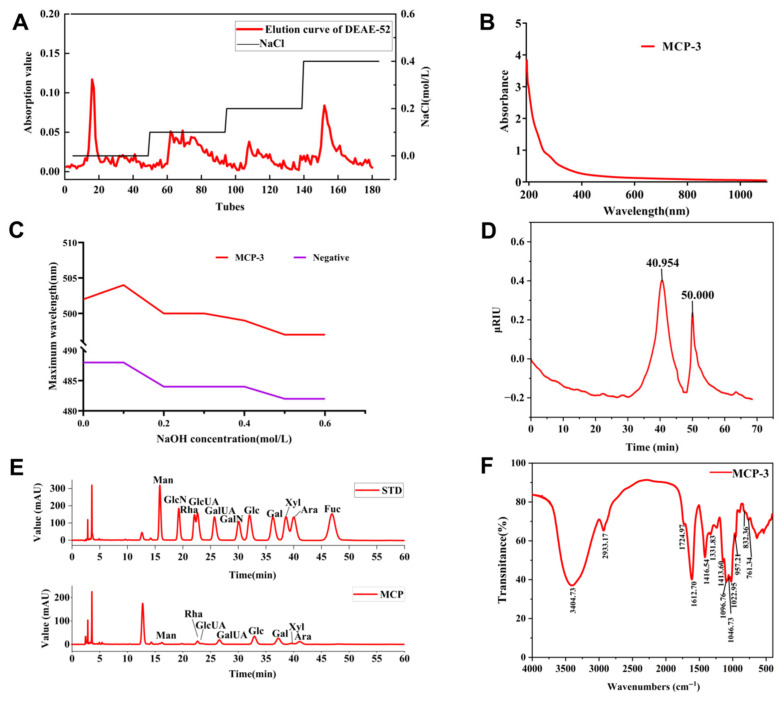
Basic structural properties of *M. chinensis* Benth polysaccharide MCP-3: (**A**) Stepwise elution curve of crude *M. chinensis* Benth polysaccharide MCP on a DEAE-52 column; (**B**) UV spectrum of MCP-3; (**C**) UV adsorption spectrum of MCP-3 in Congo Red test. Note: negative, Congo Red; (**D**) the chromatogram of molecular weight distribution of MCP-3 by HPGPC; (**E**) Monosaccharide composition of MCP-3 determined by liquid chromatography system; (**F**) The FT-IR spectrum of MCP-3 in the range of wavenumber from 4000 to 500 cm^−1^.

MCP was separated and purified using a DEAE-52 purification column. The fractions eluted with pure water and 0.1, 0.2, and 0.4 mol/L of NaCl were named MCP-C and MCP-1, MCP-2, and MCP-3, respectively. The elution curves were plotted, and the polysaccharide and protein contents were measured; the results are presented in Figure 1A and Table S3. In this study, MCP-3, after purification by Dextran 100, was further analyzed as it is one of the major components and demonstrates high activity. As shown in Figure 1B, weak absorbance was observed at 260 nm, but no obvious UV adsorption was detected at 280 nm, indicating that MCP-3 is a polysaccharide–protein complex without nucleic acids [29,40].

### 3.2. Structural Analysis and Characterization

#### 3.2.1. Stereochemical Structure

Congo Red assay was employed to tentatively explore the triple-helix structure of MCP-3. As presented in Figure 1C, the maximum absorption wavelength exhibited a red shift from 488 to 495 nm, indicating the formation of a complex between MCP-3 and Congo Red. Furthermore, the absorbance of MCP-3 gradually decreased with increasing NaOH aqueous solution from 0 to 0.5 mol/L. These results suggested that MCP-3 adopts a tightly folded triple-helix structure.

#### 3.2.2. Molecular Weight

The biological activities of polysaccharides are closely related to their molecular weight and monosaccharide composition. To determine the structure of MCP-3, its molecular weights and monosaccharide composition were analyzed. The HPGPC result, shown in Figure 1D and summarized in Table S4, showed that after purification of MCP-3 using a column packed with Dextran 100, a single peak emerged at 40.594 min, exhibiting an excellent peak shape. This result indicates that MCP-3 is likely a single-component polysaccharide. In contrast, the peak observed at 50 min was attributable to the solvent peak. The comparison of the chromatogram with the standard curve revealed that the molecular weight of MCP-3 was 16.014 kDa. These values were comparable to those of the neutral and acidic fractions separated from crude polysaccharides extracted from *M. chinensis* Benth; however, the molecular weight of MCP-3 in this study was notably lower than those reported in previous studies [21,29,40,41,42]. Enhanced antioxidant and immunomodulatory activities of low-molecular-weight polysaccharides have been well documented in the scientific literature, as compared to their high-molecular-weight counterparts [43].

#### 3.2.3. Monosaccharide Composition

As shown in the LC chromatogram (Figure 1E), a total of eight monosaccharides were identified from the MCP-3 sample, comprising mannose (Man), rhamnose (Rha), glucuronic acid (GlcA), galacturonic acid (GalA), glucose (Glc), galactose (Gal), xylose (Xyl), and arabinose (Ara), with molar mass percentage content of 3.44, 21.28, 3.52, 34.45, 25.21, 14.76, 12.12, and 10.65, respectively, indicating that MCP-3 is a hetero-polysaccharide. Low-molecular-weight heteropolysaccharides exhibit notable functional activities, such as immunomodulatory [44] and hypoglycemic effects [45]. The monosaccharide composition in our study was similar to that of MCP reported by Lin et al. [40] and Niu et al. [41], but different from those reported by Chen et al. [46] and Tang et al. [29]. These discrepancies in molecular weight and monosaccharide composition are likely attributed to differences in extraction procedures and local plant varieties.

#### 3.2.4. FT-IR Spectra

The organic functional groups of MCP-3 were analyzed by FT-IR spectroscopy, as shown in Figure 1F. Consistent with the reported findings [1,24,25,26], MCP-3 exhibited characteristic polysaccharide peaks. The adsorption peaks observed at 3404, 2933, and 1724 cm^−1^ corresponded to the O–H stretching vibration of inter- or intramolecular hydrogen bonds, the C–H stretching vibration of –CH_3_ and –CH_2_ groups, and the C=O stretching vibration of uronic acid structures, respectively. Carbonyl groups were observed near 1612 cm^−1^. Furthermore, characteristic peaks between 1450 and 1200 cm^−1^ were attributed to the stretching and angular vibrations of C–H bonds, indicating the presence of glycosidic rings. The peak at 1143 cm^−1^ was attributed to the C–O–C stretching vibration of pyranose rings. Additionally, bands at 915 and 895 cm^−1^ indicated β pyranosyl linkages, while peaks at 832 and 761 cm^−1^ indicated α pyranosyl bonds in MCP-3.

#### 3.2.5. Glycosidic Bond Composition

As shown in Table 1, the results of methylation analysis revealed that MCP-3 possesses a complex glycosidic linkage. The major linkage type in MCP-3 was 4-Gal(p)UA, which accounted for the highest molar proportion at approximately 43.52%. Following Gal(p)UA, the next most abundant linkages in MCP-3 were 4-Glcp (8.54%), 3, 4-Gal(p)-UA (6.09%), and t-Galp-UA (5.86%). In addition, the MCP-3 backbone was primarily composed of GalA glycosidic bonds, including (1→)-linked GalpA, (1→3, 4)-linked GalpA, and (1→4)-linked GalpA. The second most prevalent were Glc linkages, such as (1→)-linked Glcp, (1→4, 6)-linked Glcp, and (1→4)-linked Glcp. Other glycosidic bonds were also observed, including (1→)-linked Xylp and (1→4)-linked Xylp; (1→)-linked Galp and (1→3)-linked Galp; (1→)-linked Araf and (1→3)-linked Arap; (1→)-linked Rhap, (1→2, 4)-linked Rhap, and (1→3)-linked Rhap; (1→)-linked Fucp and (1→3, 4)-linked Fucp; (1→)-linked Glcp-UA and (1→2, 4)-linked Manp. Further structural characterization of MCP-3, especially the detailed backbone and side-chain linkages, requires NMR spectroscopy.

#### 3.2.6. NMR Characterization

**Figure 2:**
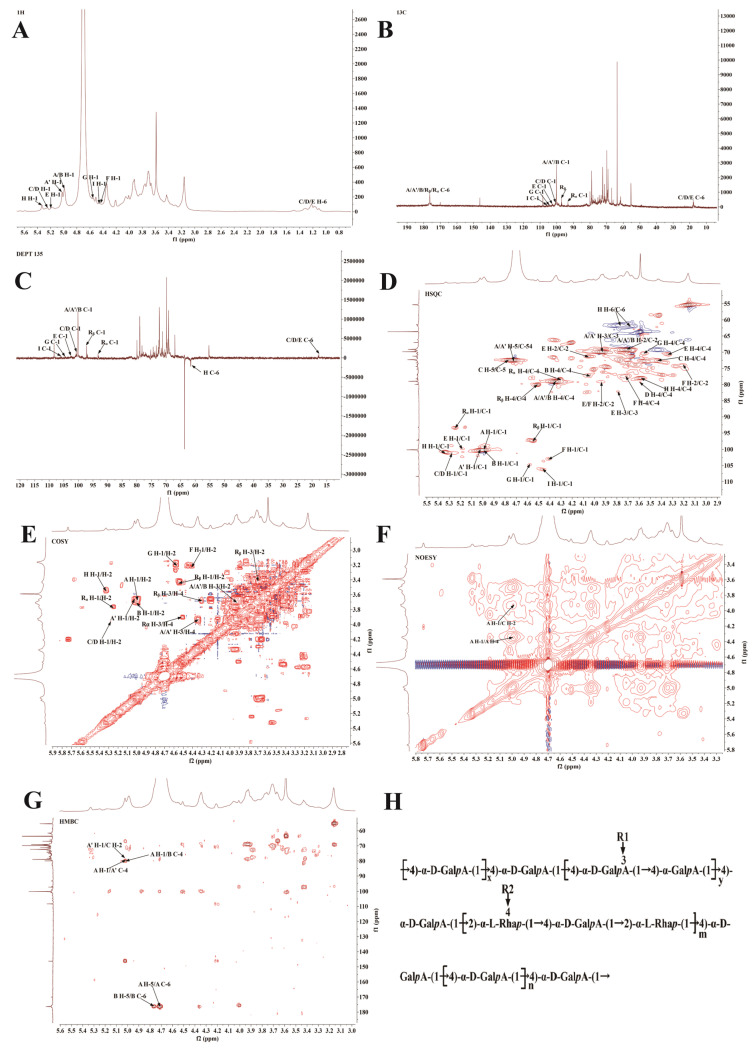
NMR spectra of *M. chinensis* Benth polysaccharide MCP-3: (**A**) ^1^H-NMR (500 MHz) spectrum; (**B**) ^13^C-NMR (125 MHz) spectrum; (**C**) DEPT-135 spectrum; (**D**) HSQC spectrum; (**E**) ^1^H-^1^H COSY spectrum; (**F**) NOESY spectrum; (**G**) HMBC spectrum; (**H**) Tentative structure of MCP-3.

The NMR spectral data of MCP-3, including ^1^H-NMR, ^13^C-NMR, and DEPT-135, and two-dimensional NMR spectra (HSQC, ^1^H-^1^H COSY, NOESY, and HMBC) are shown in Figure 2. The ^1^H-NMR spectrum of MCP-3 exhibited a range of proton resonance signals primarily concentrated between δ 3.0 and 5.5 ppm. The major end-group proton peaks were observed at δ 5.33, 5.27, 5.24, 5.18, 5.02, 4.99, 4.98, 4.57, 4.53, 4.46, and 4.41 ppm, and these peaks were concentrated between δ 4.4 and δ 5.5 ppm [47]. In addition, ring proton signals were observed in the region of δ 3.0–4.3 ppm. A distinct resonance signal between δ 1.10 and 1.25 ppm was assigned to the methyl proton (H-6) of Rha residues, confirming the presence of Rha residues in MCP-3.

In the ^13^C-NMR spectra (Figure 2B), the major anomeric carbon peaks of MCP-3 were identified in the region of δ 90–110 ppm, with distinct peaks at δ 106.20, 104.68, 102.93, 102.12, 101.11, 100.92, 100.51, 100.33, 100.23, 97.33, and 93.41 ppm. Meanwhile, the chemical shifts observed in the δ 65–85 ppm region, which migrated to the low-field region, indicated substitutions at the C2, C3, and C4 positions of sugar residues, suggesting the presence of glycosidic bonds at these positions in the MCP-3 structure. The characteristic absorption peak at δ 177.44 ppm was attributed to the C-6 carbon of unesterified GalA residues [48].

In the DEPT-135 spectrum (Figure 2C), characteristic signal peaks corresponding to C6 were observed in the high-field region of δ 60–80 ppm, indicating the presence of C6-linked residues in MCP-3. Furthermore, the shifts of glycosidic bond signals to low-field regions suggest the presence of substitutions [49]. According to the HSQC spectrum, a prominent anomeric proton signal was observed at δ 5.27 ppm, with its corresponding anomeric carbon signal at δ 101.11 ppm (Figure 2D). The distinguished signals of the anomeric proton were assigned to the H1, H2, H3, and H4 positions of glycosyl residue C, with chemical shifts at δ 5.27, 3.95, 3.77, and 3.43 ppm (Figure 2E). The NOESY spectrum (Figure 2F) showed that the anomeric proton signal δ 5.27 ppm correlated with signals at δ 3.95, 3.77, and 3.43 ppm, which indicated that the peaks for C5 related to H5 (δ 3.67 ppm) were δ 69.36 ppm, for C6 corresponding to H6 δ 1.20 ppm [50]. Consequently, these peaks were attributed to the glycosidic bond →2)-α-L-Rhap-(1→(C). Similarly, the assignment of chemical shifts for the glycosidic bonds in MCP-3 is summarized in Table 2 based on the correlated signals observed in the HSQC, COSY, and NOESY spectra.

The linkage sequence of the glycosidic residues in MCP-3 was deduced from the HMBC spectrum (Figure 2G). A correlation peak between the anomeric carbon (at δ 79.27 ppm) of residue A and its H4 proton at δ 4.99 ppm was observed, suggesting that two →4)-α-GalpA-(1→ units were linked through the C4 position. An interconnection between the anomeric proton (δ 4.99 ppm) of residue A and the C4 carbon (δ 79.20 ppm) of residue B was observed, indicating the linkage mode →4)-α-GalpA-(1→3, 4)-α-GalpA- (1→ [51,52]. Furthermore, a correlation peak between the anomeric proton of A’ (δ 5.02 ppm) and the anomeric carbon of C (δ 79.20 ppm) indicated a linkage at the C2 position, corresponding to →4)-α-GalpA-(1→2)-α-l-Rhap-(1→). As shown in the NOESY spectrum (Figure 2F), a correlation peak was observed between glycosidic residue A (δ 4.99 ppm) and residue C (δ 3.95 ppm), indicating a linkage of →4) -α-Galpa-(1→2)-α-l-Rhap-(1→) at the C2 position. Moreover, the correlation between H1 (δ 4.99 ppm) and H4 (δ 4.35 ppm) of residue A indicated a self-linkage of →4)-α-GalpA-(1→ at the C4 position [53,54].

Based on the combined results of NMR spectroscopy, methylation analysis, and monosaccharide composition, the backbone structure of MCP-3 was inferred to comprise of →4)-α-D-GalpA-6-(1→4)-α-GalpA-(1→ and →4)-α-D-GalpA-6-(1→2)-α-L-Rhap-(1→4)-α-D-GalpA-6-(1→2,4)-α-L-Rhap-(1→ [52]. The branched chains were connected to the backbone through the O-3 position of arabinoxylan fragments, the O-4 position of →3)-α-L-Rhap-(1→, →4)-α-D-Glcp-(1→, β-D-Galp-(1→, and other glycosidic residues [46,47,48,49,50,51,52,53,54,55,56,57,58,59]. The proposed structure of MCP-3 is shown in Figure 2H.

#### 3.2.7. Morphological Characteristics

**Figure 3:**
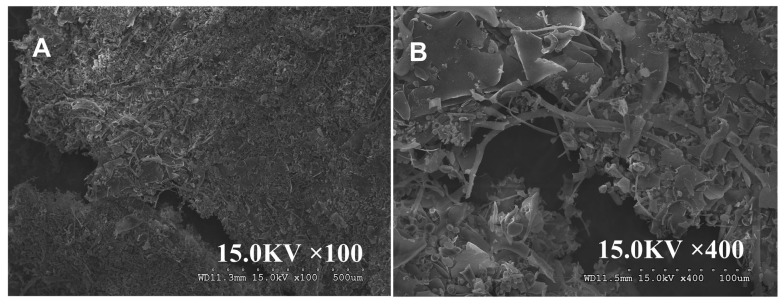
Microstructure images of *M. chinensis* Benth polysaccharide MCP-3: (**A**) The morphology of MCP-3 at 100×, scalebar is 500 μm; (**B**) The morphology of MCP-3 at 400×, scalebar is 100 μm.

The image of MCP is shown in Figure S2. MCP exhibited a sheet-like structure with internal pores at 100× magnification. At 400× magnification, its surface appeared uniform, smooth, and compact, with neat edges, thick walls, and some cable-like structures. In contrast, MCP exhibits a denser and more finely fragmented structure than that of MCP at 100× magnification (Figure 3). At 400× magnification, MCP-3 displays cord-like and paper-like morphologies with smooth, compact surfaces, neat edges, and thick walls. These structural features are likely attributed to the hydrophobic interactions and hydrogen bonding among the carboxyl and hydroxyl groups in the polysaccharide molecules, which promote the ordered aggregation of polymer chains.

### 3.3. In Vitro Antioxidant Ability

**Figure 4:**
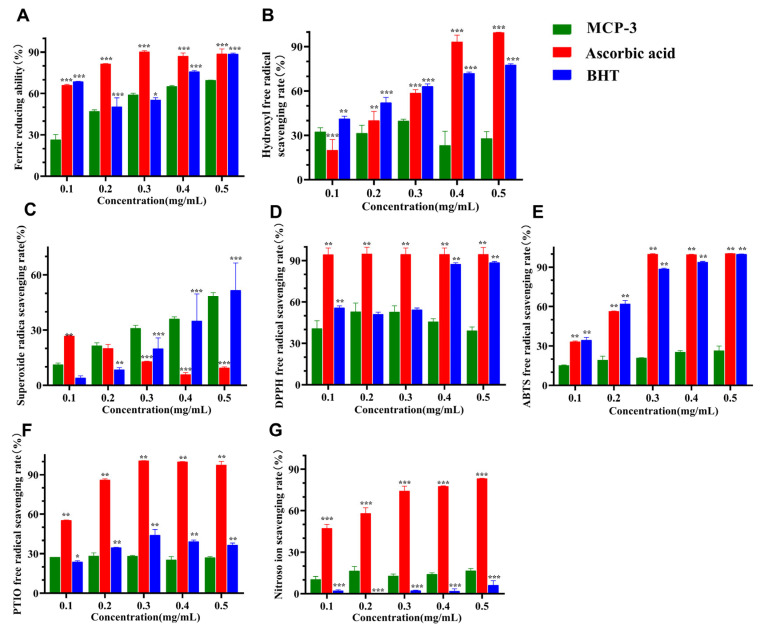
In vitro antioxidative abilities of *M. chinensis* Benth polysaccharide MCP-3 determined by microplate spectrophotometer: (**A**) Ferric reducing ability; (**B**) Hydroxyl free radical scavenging ability; (**C**) Superoxide anion free radical scavenging ability; (**D**) DPPH free radical scavenging ability; (**E**) ABTS free radical scavenging ability; (**F**) PTIO free radical scavenging ability; (**G**) Nitroso ion scavenging ability. Ascorbic acid vs. MCP-3: *** *p* < 0.001; ** *p* < 0.01; BHT vs. MCP-3: *** *p* < 0.001; ** *p* < 0.01; * *p* < 0.05.

The in vitro antioxidant abilities of MCP-3 in eliminating free radicals are shown in Figure 4 and Table S5. Compared with the positive controls (ascorbic acid and BHT), MCP-3 demonstrated ferric reducing activity comparable to those reported in a previous study [29]. This activity may be attributed to its capacity to stabilize reactive free radicals and terminate free radical chain reactions by donating electrons to the radicals. The maximum scavenging rate for hydroxyl radicals (39.7 ± 1.2%) was observed at a relatively lower concentration (0.3 mg/mL), which was similar to the scavenging ability reported by Tang et al. [29]; nonetheless, the scavenging ability of hydroxyl radicals in this study was mildly lower than that in previous study [40]. These results indicate that MCP-3 is a potential scavenger capable of eliminating excess hydroxyl radicals, which could contribute to severe cellular and tissue damage. The ferric-reducing ability of MCP-3 also exhibited a trend similar to its superoxide anion free-radical scavenging ability, with the scavenging rate increasing to 69.6 ± 0.1% with an increase in polysaccharide concentration, indicating a dose–response relationship. In the superoxide radical-scavenging assay, the elimination rate increased with MCP-3 concentration, reaching 48.3 ± 2.1%, indicating a dose-dependent effect. The scavenging ability of MCP-3 was higher than that of ascorbic acid but lower than that of BHT. These results indicated that MCP-3 demonstrated a moderate efficacy in scavenging superoxide anion radicals, contributing to reducing ROS levels and mitigating ROS-induced DNA damage. Nonetheless, the scavenging ability of MCP-3 was slightly lower than that reported by Lin et al. [40]. In this study, MCP-3 solutions at different concentrations included notable free radical-scavenging effects. Specifically, MCP-3 demonstrated moderate DPPH radical-scavenging ability, reaching a maximum value of 53.0 ± 6.2% at a concentration of 0.2 mg/mL. However, no significant increase (*p* > 0.05) in scavenging ability was observed with increasing polysaccharide concentration. MCP-3 demonstrated considerable DPPH radical-scavenging ability, consistent with findings from previous studies [29,40]. However, no significant differences (*p* > 0.05) were observed in ABTS and PTIO scavenging abilities across different MCP-3 concentrations. The highest scavenging rates of 26.3 ± 2.9% and 28.3 ± 2.4% were observed at 0.5 and 0.2 mg/mL, respectively. Similarly, nitroso ion inhibition was independent of the polymer concentration, maintaining a consistent scavenging ability of approximately 16.7 ± 1.6%.

The in vitro antioxidant assays demonstrated that MCP-3 exhibited satisfactory scavenging and reducing abilities against certain free radicals such as DPPH, superoxide, and hydroxyl radicals; nonetheless, the elimination capacities of MCP-3 for nitroso ion, ABTS, and PTIO radicals were significantly lower, indicating that MCP-3 does not possess broad-spectrum antioxidant ability. The antioxidant activity of MCP-3 may be attributed to its low molecular weight and the presence of uronic acids with reducing ability and free hydroxyl groups [53]. These findings can enhance our understanding of the oxidation resistance of MCP-3 and further support the hypothesis that MCP-3 could serve as an effective antioxidant. Therefore, we investigated whether MCP-3 exerts a protective effect against oxidative stress in intestinal epithelial cells caused by food contaminants to explore its potential application in managing enteric diseases, such as inflammatory bowel disease, duodenal ulcer, and ulcerative colitis.

### 3.4. Ameliorative Effects of MCP-3 on DON-Induced Oxidative Stress

DON, a highly containing food contaminant, primarily targets the intestine, where it induces considerable toxicity by disrupting the intestinal structure, impairing the epithelial barrier, and compromising the mucosal immune system through redox homeostasis imbalance [12]. To evaluate protective strategies against DON-induced intestinal damage, MCP-3 was selected for investigating its potential ameliorative effects against DON-induced oxidative stress in Caco-2 and NCM460 cells.

#### 3.4.1. Effects of MCP-3 and DON on Cell Proliferation in Caco-2 and NCM460 Cells

As presented in Figure S3, the results of MTT assays indicated that there were no significant differences in Caco-2 cell viability (*p* > 0.05) observed between the control group and treatment groups when MCP-3 concentration was increasing from 3.91 to 1000 μg/mL, whereas inhibition of proliferation was observed in NCM460 cells when the treatment dose of MCP-3 was set to 1000 μg/mL. Consequently, three appropriate treatment levels were confirmed for the follow-up study on the ameliorative effects of MCP-3, namely, low level (25 μg/mL), medium level (100 μg/mL), and high level (400 μg/mL). In contrast, DON exerted a significant cytotoxicity effect (*p* < 0.05) on Caco-2 and NCM460 cells, indicating that the inhibitory effects of DON on proliferation were concentration-dependent. DON concentrations beyond 3 µg/mL (Caco-2 cells) and 0.064 µg/mL (NCM460 cells) exhibited a significant difference in viability compared with the control group. However, Caco-2 cells maintained viability above 80% at a DON concentration of 5 µg/mL, whereas NCM460 cells exhibited viability over 80% when the DON concentration was at 0.32 µg/mL. Thus, it was decided to use 5 µg/mL of DON as the treatment concentration for Caco-2 cells and 0.32 µg/mL for NCM460 cells. Due to the exposure dose of DON in this study being confirmed based on the cell viability assay, the exposure dose in further in vivo studies should be selected at a level closer to the real dietary exposure of the population to eliminate the limitations [14].

#### 3.4.2. Effects of MCP-3 on DON-Induced Oxidative Stress

**Figure 5:**
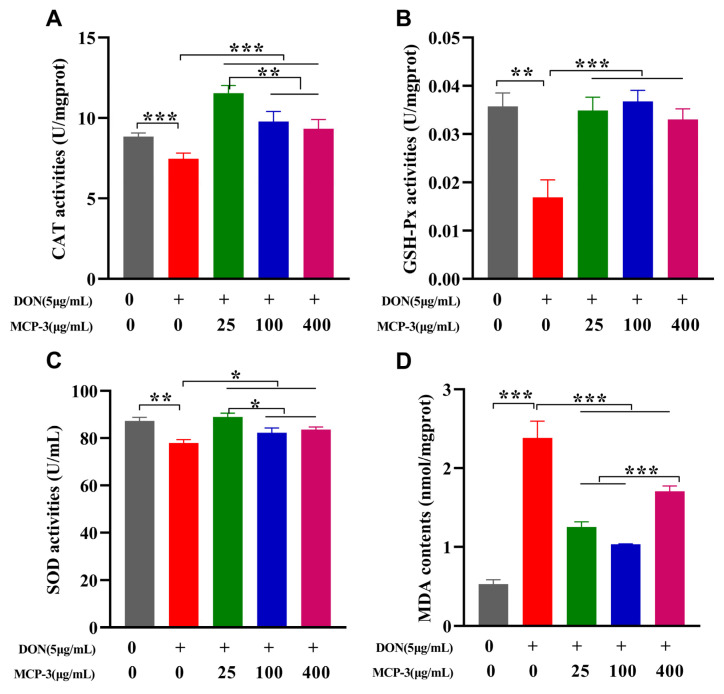
*M. chinensis* Benth polysaccharide MCP-3 enhanced antioxidant enzyme activities and decreased MDA accumulation of DON-treated Caco-2 cells: (**A**) CAT; (**B**) GSH-Px; (**C**) SOD; (**D**) MDA. Note: Control: 0 µg/mL DON + 0 µg/mL MCP-3; Model: 0.5 µg/mL DON + 0 µg/mL MCP-3; Low-dose treatment group: 5 µg/mL DON + 25 µg/mL MCP-3; Medium-dose treatment group: 5 µg/mL DON + 100 µg/mL MCP-3; High-dose treatment group: 5 µg/mL DON + 400 µg/mL MCP-3. *** *p* < 0.001; ** *p* < 0.01; * *p* < 0.05.

**Figure 6:**
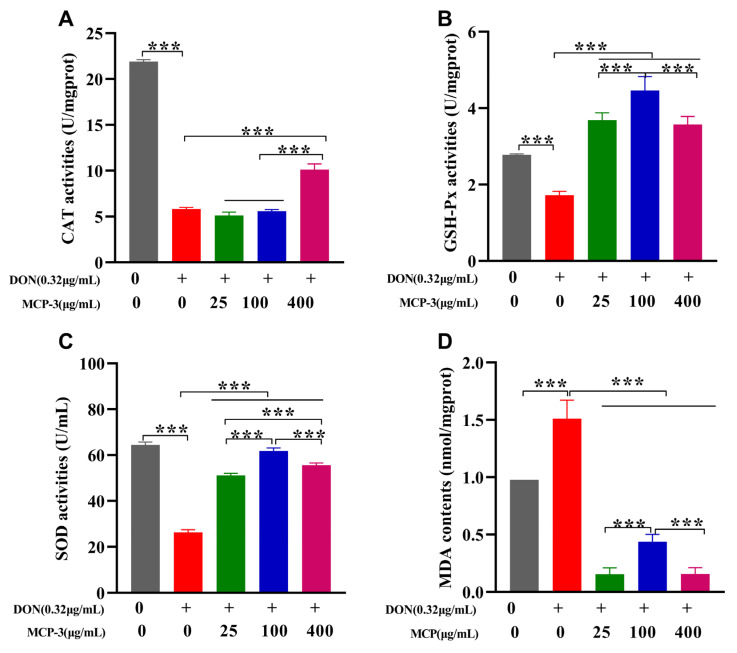
*M. chinensis* Benth polysaccharide MCP-3 enhanced antioxidant enzyme activities and decreased MDA accumulation of DON-treated NCM460 cells: (**A**) CAT; (**B**) GSH-Px; (**C**) SOD; (**D**) MDA. Note: Control: 0 µg/mL DON + 0 µg/mL MCP-3; Model: 0.32 µg/mL DON + 0 µg/mL MCP-3; Low-dose treatment group: 0.32 µg/mL DON + 25 µg/mL MCP-3; Medium-dose treatment group: 0.32 µg/mL DON + 100 µg/mL MCP-3; High-dose treatment group: 0.32 µg/mL DON + 400 µg/mL MCP-3. *** *p* < 0.001.

As presented in Figure 5 and Figure 6 and Table S5, a significant reduction (*p* < 0.05) in CAT, SOD, and GSH-Px activities was observed in the DON group compared to the control, indicating that DON markedly suppressed antioxidant enzyme activities in intestinal epithelial cells. Additionally, a notable increase in the oxidative stress marker MDA (*p* < 0.05) was observed in both the cell lines, suggesting that DON exposure induced oxidative stress, potentially leading to serious inflammation or intestinal injury [16].

Owing to the remarkable ameliorative effect of MCP-3, all three treatment groups showed significant recovery (*p* < 0.05) in CAT, SOD, and GSH-Px activities in both Caco-2 and NCM460 cells, which exhibited the excellent cytoprotective potential of MCP-3 against DON-induced oxidative stress. The MCP-3 concentration of 25 µg/mL was more effective (*p* < 0.05) in enhancing the antioxidant enzyme activities in Caco-2 cells, while the optimal concentration in NCM460 cells was 100 µg/mL, except for CAT activity. These findings were consistent with the results of in vitro free radical scavenging assays and align with the results reported by Tang et al. [29] and Lin et al. [40]. A sharp decrease (*p* < 0.05) in MDA accumulation was observed in Caco-2 cells treated with 100 µg/mL MCP-3 and in NCM460 cells treated with 400 µg/mL MCP-3 (Figure 5D and Figure 6D), indicating that MCP-3 significantly improved (*p* < 0.05) DON-induced lipid oxidation in enterocytes.

Although GSH-Px activity recovery and MDA elimination in Caco-2 cells were enhanced at 100 µg/mL of MCP-3, the improvements showed no significant difference from the results observed at 25 µg/mL of MCP-3. Accordingly, the optimal MCP-3 concentration for ameliorating DON-induced oxidative stress was 25 µg/mL MCP-3 in Caco-2 cells and 100 µg/mL in NCM460 cells.

#### 3.4.3. Effects of MCP-3 on ROS Generation Induced by DON

**Figure 7:**
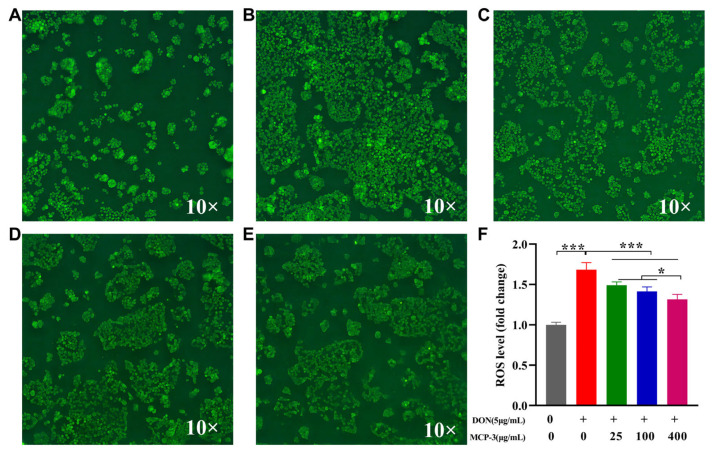
*M. chinensis* Benth polysaccharide MCP-3 decreased intracellular ROS in DON-treated Caco-2 cells: (**A**) Control: 0 µg/mL DON + 0 µg/mL MCP-3; (**B**) Model: 5 µg/mL DON + 0 µg/mL MCP-3; (**C**) Low-dose treatment group: 5 µg/mL DON + 25 µg/mL MCP-3; (**D**) Medium-dose treatment group: 5 µg/mL DON + 100 µg/mL MCP-3; (**E**) High-dose treatment group: 5 µg/mL DON + 400 µg/mL MCP-3; (**F**) Fold change of ROS level. Note: *** *p* < 0.001; * *p* < 0.05.

**Figure 8:**
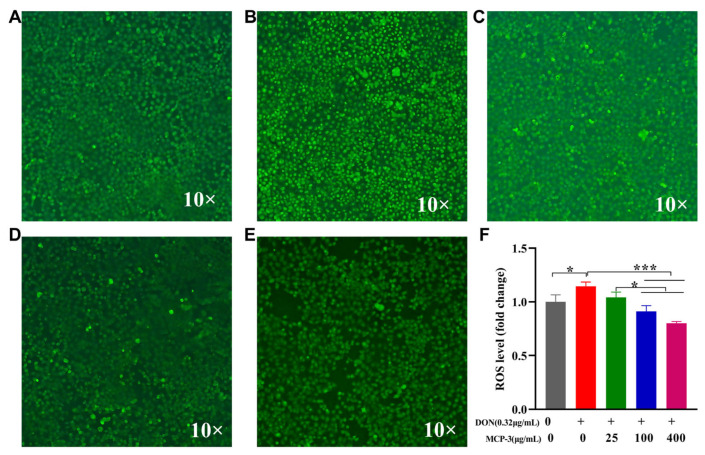
*M. chinensis* Benth polysaccharide MCP-3 decreased intracellular ROS in DON-treated NCM460 cells: (**A**) Control: 0 µg/mL DON + 0 µg/mL MCP-3; (**B**) Model: 0.32 µg/mL DON + 0 µg/mL MCP-3; (**C**) Low-dose treatment group: 0.32 µg/mL DON + 25 µg/mL MCP-3; (**D**) Medium-dose treatment group: 0.32 µg/mL DON + 100 µg/mL MCP-3; (**E**) High-dose treatment group: 0.32 µg/mL DON + 400 µg/mL MCP-3; (**F**) Fold change of ROS level. Note: *** *p* < 0.001; * *p* < 0.05.

ROS is a normal oxygen metabolism that is closely associated with the synthesis of a series of inflammatory factors that influence cell signaling and homeostasis. As shown in Figure 7 and Figure 8, different MCP-3 concentration treatments could remarkably reduce (*p* < 0.05) ROS generation compared with DON in Caco-2 and NCM460 cells. Moreover, the production of ROS in the MCP-3 treatment group was notably decreased (*p* < 0.05) in a dose-dependent manner, which is consistent with the previously reported inhibitory effects of MCP-3 on ROS production in H_2_O_2_-induced LO2 cells injury [46]. Fluorescence microscopic images also showed the inhibitory effects of MCP-3 on the oxidative stress of enterocytes induced by DON. These results indicated that MCP-3 plays a protective role against oxidative damage by reducing the accumulation of intracellular ROS in a dose-dependent manner.

### 3.5. Effects of MCP-3 on Inflammatory Cytokines Induced by DON

**Figure 9:**
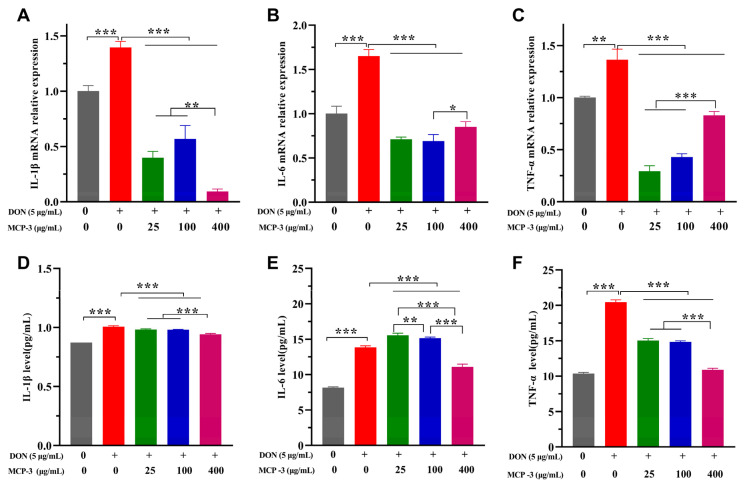
*M. chinensis* Benth polysaccharide MCP-3 decreased the mRNA relative expression and secretion level of pro-inflammatory cytokines in Caco-2 cells: mRNA relative expressions: (**A**) IL-1β; (**B**) IL-6; (**C**) TNF-α; secretion level: (**D**) IL-1β; (**E**) IL-6; (**F**) TNF-α. Note: *** *p* < 0.001; ** *p* < 0.01; * *p* < 0.05.

**Figure 10:**
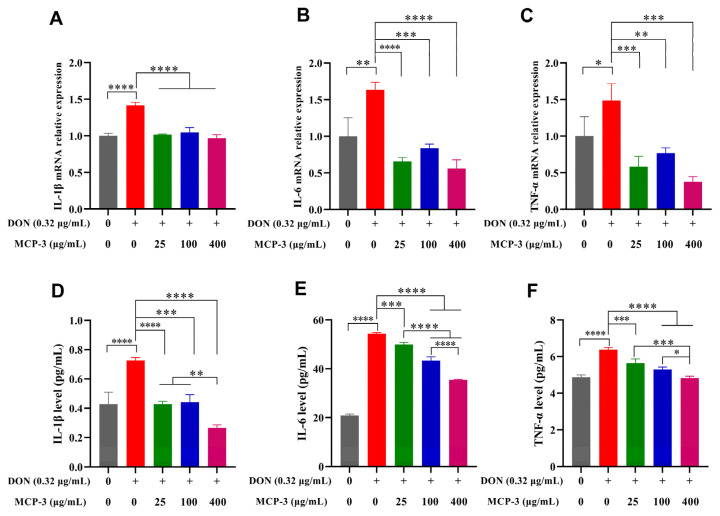
*M. chinensis* Benth polysaccharide MCP-3 decreased the mRNA relative expression and secretion level of pro-inflammatory cytokines in NCM460 cells: mRNA relative expressions: (**A**) IL-1β; (**B**) IL-6; (**C**) TNF-α; secretion level: (**D**) IL-1β; (**E**) IL-6; (**F**) TNF-α. Note: **** *p* < 0.0001; *** *p* < 0.001; ** *p* < 0.01; * *p* < 0.05.

The expressions of IL-1β, IL-6, and TNF-α are critical indicators for the evaluation of the inflammatory regulation of enterocytes caused by external simulation [60,61]. The relative expression of genes was evaluated via RT-qPCR, as shown in Figure 9 and Figure 10. DON sharply increased the gene expression of three cytokines in Caco-2 and NCM460 cells. Thanks to the remarkable anti-inflammatory effects of MCP-3, the mRNA expression levels of three cytokines were strikingly lower than those of the DON group in two enterocytes, even the blank control group (*p* < 0.05). It is indicated that MCP-3 first downregulates the cytokine expression at the gene level, effectively inhibiting the excessive inflammation triggered by DON.

To further evaluate the regulation of MCP-3 on the release of IL-1β, IL-6, and TNF-α, the cellular secretion of these three cytokines was investigated via the ELISA method. As illustrated in Figure 9 and Figure 10, significant oversecretion of cytokines (*p* < 0.05) was observed in the DON treatment group, which indicated that excessive inflammation is detrimental to cells. The effect of MCP-3 on the cytokine generation in this study was found to be considerably lower (*p* < 0.05) than that of the DON group, indicating that MCP-3 regulated the generation of proinflammatory mediators; however, MCP-3 could not recover the cytokine level to the normal state. Taken together, it is evident that MCP-3 exerted a favorable inhibitory effect on the excessive expression of inflammatory factors induced by DON. Compared with plant compounds reported in the literature, such as lycopene [7], melatonin [10], and lauric acid [11], the polysaccharide MCP-3 extracted from *M. chinensis* Benth showed comparable protective effects against mycotoxin-induced oxidative stress in intestinal cells.

## 4. Conclusions

In summary, a hetero-polysaccharide of *M. chinensis* Benth, MCP-3, was extracted and purified successfully; its structural characteristic was systematically elucidated using multi-dimensional characterization methods. Structural analysis revealed that MCP-3 mainly consisted of Man, Rha, GlcA, GalA, Glc, Gal, Xyl, and Ara with a molecular weight of 16.014 kDa, having a backbone of →4)-α-D-GalpA-6-(1→4)-α-GalpA-(1→ and →4)-α-D-GalpA-6-(1→2)-α-L-Rhap-(1→4)-α-D-GalpA-6-(1→2,4)-α-L-Rhap-(1→ linkages. According to the results of in vitro antioxidation assays, MCP-3 possessed appreciable antioxidant ability, particularly in scavenging DPPH, superoxide, and hydroxyl radicals. Most importantly, this study provides compelling evidence that MCP-3 exerts a protective effect against DON-induced oxidative injury in human intestinal epithelial cells by modulating inflammatory homeostasis. It is well known that this is the first study to attempt to elucidate the potential mechanism underlying the protective role of MCP-3 in counteracting the intestinal toxicity induced by DON, thereby offering a novel direction for further understanding the beneficial effects of MCP-3. However, further in vivo studies are needed to elucidate the specific protective mechanisms of MCP-3 against enterotoxicity induced by the actual dietary exposure of DON, and clarify the relationship between MCP-3 activity, molecular structure, and conformation.

## Figures and Tables

**Table 1:** Molar ratio of glycosidic linkages in *M. chinensis* Benth polysaccharide MBP-3 by methylation analysis using GC-MS.

Retention Time (min)	Methylated Sugar	Mass Fragments (*m*/*z*)	Linkage Pattern	Molar Ratio (%)
11.692	1,4-di-*O*-acetyl-2,3,5-tri-*O*-methyl arabinitol	32, 43, 60, 71, 87, 102, 113, 118, 129, 161	Ara*f*-(1→	3.22
12.734	1,5-di-*O*-acetyl-2,3,4-tri-*O*-methyl xylitol	32, 43, 59, 72, 89, 102, 118, 131, 162	Xyl*p*-(1→	4.98
12.734	1,5-di-*O*-acetyl-6-deoxy-2,3,4-tri-*O*-methyl rhamnitol	32, 40, 43, 60, 72, 89, 102, 118, 131, 162, 175	Rha*p*-(1→	2.80
13.607	1,5-di-*O*-acetyl-6-deoxy-2,3,4-tri-*O*-methyl fucitol	32, 40, 43, 60, 74, 87, 101, 118, 129	Fuc*p*-(1→	1.67
15.464	1,3,5-tri-*O*-acetyl-2,4-di-*O*-methyl arabinitol	32, 43, 60, 72, 89, 100, 115, 131, 190	→3)-Ara*p*-(1→	1.57
15.496	1,2,5-tri-*O*-acetyl-6-deoxy-3,4-di-*O*-methyl rhamnitol	32, 40, 43, 60, 87, 102, 118, 129, 189	→2)-Rha*p*-(1→	3.16
15.638	1,4,5-tri-*O*-acetyl-2,3-di-*O*-methyl xylitol	32, 40, 43, 59, 72, 89, 101, 118, 128, 131, 202, 234	→4)-Xyl*p*-(1→	2.37
15.910	1,3,5-tri-*O*-acetyl-6-deoxy-2,4-di-*O*-methyl rhamnitol	32, 40, 43, 60, 87, 100, 118, 129, 132, 189	→3)-Rha*p*-(1→	2.11
16.777	1,5-di-*O*-acetyl-2,3,4,6-tetra-*O*-methyl glucitol	32, 40, 43, 60, 73, 87, 102, 118, 129, 145, 162, 207	Glc*p*-UA-(1→	0.82
16.777	1,5-di-*O*-acetyl-2,3,4,6-tetra-*O*-methyl glucitol	32, 43, 73, 87, 102, 118, 131, 147, 162, 207	Glc*p*-(1→	1.30
17.469	1,5-di-*O*-acetyl-2,3,4,6-tetra-*O*-methyl galactitol	32, 43, 60, 87, 99, 113, 118, 129, 173, 275	Gal*p*-UA-(1→	5.86
17.469	1,5-di-*O*-acetyl-2,3,4,6-tetra-*O*-methyl galactitol	32, 40, 43, 60, 88, 101, 117, 130, 143, 190, 203	Gal*p*-(1→	4.37
17.799	1,3,4,5-tetra-*O*-acetyl-6-deoxy-2-*O*-methyl fucitol	32, 40, 43, 60, 88, 100, 130, 163, 190, 207	→3,4)-Fuc*p*-(1→	1.14
18.394	1,2,4,5-tetra-*O*-acetyl-6-deoxy-3-*O*-methyl rhamnitol	32, 40, 43, 60, 87, 99, 118, 129, 157, 171, 231	→2,4)-Rha*p*-(1→	2.03
19.688	1,3,5-tri-*O*-acetyl-2,4,6-tri-*O*-methyl glucitol	32, 40, 43, 60, 71, 87, 101, 118, 129, 161, 207, 234	→3)-Glc*p*-(1→	0.79
19.810	1,4,5-tri-*O*-acetyl-2,3,6-tri-*O*-methyl galactitol	32, 43, 47, 87, 102, 115, 118, 129, 144, 162, 175, 235	→4)-Gal*p*-UA-(1→	43.52
20.037	1,4,5-tri-*O*-acetyl-2,3,6-tri-*O*-methyl glucitol	32, 43, 59, 71, 87, 102, 113, 118, 129, 142, 162, 173, 233	→4)-Glc*p*-(1→	8.54
20.309	1,3,5-tri-*O*-acetyl-2,4,6-tri-*O*-methyl galactitol	32, 40, 43, 60, 71, 87, 101, 118, 129, 161, 174, 190, 234	→3)-Gal*p*-(1→	1.38
21.958	1,3,4,5-tetra-*O*-acetyl-2,6-di-*O*-methyl galactitol	32, 43, 47, 59, 87, 118, 131, 145, 160, 186, 307	→3,4)-Gal*p*-UA-(1→	6.09
22.560	1,2,4,5-tetra-*O*-acetyl-3,6-di-*O*-methyl mannitol	32, 40, 43, 60, 88, 99, 113, 130, 140, 190, 207, 233	→2,4)-Man*p*-(1→	1.78
23.323	1,4,5,6-tetra-*O*-acetyl-2,3-di-*O*-methyl glucitol	32, 40, 43, 60, 69, 85, 102, 118, 127, 159, 207, 261	→4,6)-Glc*p*-(1→	0.52

**Table 2:** ^1^H and ^13^C-NMR chemical shift data (δ, ppm) for *M. chinensis* Benth polysaccharide MCP-3 determined in D_2_O at 70 °C.

Glycosyl Residues		Chemical Shift (ppm)							
			1	2	3	4	5	6a	6b
A	→4)-α-Gal*p*A-(1→	H	4.99	3.69	3.93	4.35	4.72	ns ^a^	
		C	100.23	69.36	69.89	79.27	72.41	176.44	
A’	→4)-α-Gal*p*A-(1→	H	5.02	3.67	3.93	4.35	4.71	ns	
		C	100.51	71.28	69.85	79.27	72.52	176.44	
B	→3,4)-α-Gal*p*A-(1→	H	4.98	3.71	4.04	4.35	4.76	ns	
		C	100.33	69.36	77.26	79.27	72.23	176.23	
C	→2)-α-L-Rha*p*-(1→	H	5.27	3.95	3.77	3.43	3.67	1.20	
		C	101.11	79.2	72.41	72.52	69.36	17.85	
D	→2,4)-α-L-Rha*p*-(1→	H	5.27	3.95	3.78	3.59	3.84	1.20	
		C	101.11	79.20	72.43	79.23	70.25	17.82	
E	→3)-α-L-Rha*p*-(1→	H	5.18	4.04	3.78	3.35	3.73	1.20	
		C	102.12	71.23	82.27	70.61	ND	17.82	
F	→4)-β-D-Xyl*p*-(1→	H	4.41	3.20	3.48	3.71	3.29	4.02	
		C	102.93	74.11	74.92	77.33	63.83	ns	
G	β-D-Xyl*p*-(1→	H	4.57	3.20	3.39	3.55	3.29	4.02	
		C	104.68	74.11	76.99	70.35	63.83	ns	
H	→4)-α-D-Glc*p*-(1→	H	5.33	3.57	3.89	3.59	3.67	3.77	3.69
		C	100.92	72.75	74.27	77.87	71.00	61.81	
I	β-D-Galp-(1→	H	4.46	3.55	3.63	3.97	3.63	ns	
		C	106.20	ns	73.79	69.47	76.21	ns	
R_α_	→4)-α-D-Gal*p*A	H	5.24	3.77	3.93	4.49	4.36	ns	
		C	93.41	72.36	69.87	80.13	71.55	176.45	
R_β_	→4)-β-D-Gal*p*A	H	4.53	3.44	3.69	4.30	3.99	ns	

^a^ ns means not significant. H1 (δ 4.99 ppm) and H4 (δ 4.35 ppm) of residue A indicated a self-linkage of →4)-α-GalpA-(1→ at the C4 position.

## Data Availability

Data are contained within the article.

## Supplementary Materials

The following supporting information can be downloaded at: https://www.mdpi.com/article/10.3390/nu17162592/s1, Figure S1: Microstructure of MCP on magnification of 100× and 400×; Figure S2: Total ion chromatogram of the methylation analysis of MCP-3; Figure S3: Effects of MCP-3 and DON on viabilities of Caco-2 and NCM460 cells: (A) MCP-3 on Caco-2 cells; (B) DON on Caco-2 cells; (C) MCP-3 on NCM460 cells; (D) DON on NCM460 cells; Table S1: Reaction program of real-time PCR for mRNA relative expression of inflammatory factors; Table S2: Sequences of primers used in RT-qPCR analysis; Table S3: Physicochemical properties of MCP fractions; Table S4: Molecular weight of MCP and MCP-3; Table S5: Comparison of the isolation, characterization, and ameliorative effects of *M. chinensis* Benth polysaccharide against oxidative stress with the results reported in the published literatures. Reference [62] is cited in the supplementary materials.

## Abbreviations

The following abbreviations are used in this manuscript:

ABTS	2,2′-Azinobis-(3-ethylbenzthiazoline-6-sulphonate)
ATCC	American Type Culture Collection
BCA	Bicinchoninic acid
BHT	Butylated hydroxytoluene
CAT	Catalase
DCFH-DA	Dichlorodihydrofluorescein diacetate
DEAE-52	Diethylaminomethyl cellulose-52
DMEM	Dulbecco’s Modified Eagle’s Medium
DEPT-135	Distortionless enhancement by polarization transfer 135
DON	Deoxynivalenol
DPPH	2,2-Diphenyl-1-picrylhydrazyl
EMEM	Eagle’s Minimum Essential Medium
FBS	Fetal bovine serum
FT-IR	Fourier transform infrared spectroscopy
GSH-Px	Glutathione peroxidase
^1^H–^1^H COSY	Correlated spectroscopy ()
HMBC	Heteronuclear multiple bond correlation spectroscopy
HSQC	Heteronuclear single quantum coherence spectroscopy
MCP-3	*Mesona chinensis* Benth polysaccharide-3
MDA	Malondialdehyde
MTT	5-Diphenyltetrazolium
NMR	Nuclear magnetic resonance
NOESY	Nuclear overhauser effect spectroscopy
PTIO	2-Phenyl-4,4,5,5-tetramethylimidazoline-3-oxide-1-oxyl
ROS	Reactive oxygen species
SEM	Scanning electron microscope
SOD	Superoxide dismutase
TMSP	3-(Trimethylsilyl) propionic acid-d4 sodium salt
