# Supplementary material for: Structural Characterization and Ameliorative Effects of Mesona chinensis Benth Polysaccharide Against Deoxynivalenol-Induced Oxidative Stress in Intestinal Epithelial Cells
Source: Nutrients. 2025 Aug 9;17(16):2592. doi: 10.3390/nu17162592 (PMC12389029; doi:10.3390/nu17162592)
Supplement: Supplementary file 1 [file nutrients-17-02592-s001.zip › nutrients-3739027-supplementary.pdf]

Supplemental Materials

Structural Characterization and Ameliorative Effects of *Mesona chinensis* Benth Polysaccharide Against Deoxynivalenol-Induced Oxidative Stress in Intestinal Epithelial Cells

Ai-Hua Zhong [a, b, 1], Qiu-Yun Li [a, 1], Hua Su [a], Li-Jun Huang [a], Quan Zhou [c], Xiao-Dan Wang [d], Jia Song [a], Yong-Ning Wu [a, d], Xing-Fen Yang [a], Wei-Liang Wu [a, *]

[a] *Food Safety and Health Research Center, NMPA Key Laboratory for Safety Evaluation of Cosmetics, Guangdong-Hongkong-Macao Joint Laboratory for Contaminants Exposure and Health, Guangdong Provincial Key Laboratory of Tropical Disease Research, School of Public Health, Southern Medical University, Guangzhou 510515, P. R. China*

[b] *Public Health Service Center, Bao'an District, Shenzhen, Shenzhen 5181260, PR China*

[c] *Guangzhou Center for Food and Drug Evaluation, Guangzhou 510642, China*

[d] *NHC Key Laboratory of Food Safety Risk Assessment, Chinese Academy of Medical Science (2019RU014), China National Center for Food Safety Risk Assessment, Beijing 100022, P. R. China*

[1] Co-first authors who contributed equally to this work

*** Corresponding authors:** *Wei-Liang Wu, Associate Professor*

1. Methods

1.1. Extraction procedure for Mesona chinensis Benth polysaccharide (MCP)

The extraction process of MCP was performed according to Huang et al. [1] with minor modification. The *Mesona chinensis* Benth (*M. chinensis* Benth) powder was immersed into a clean round bottomed flask with reflux condensation filled with 95% ethanol solution (v/v) in a ratio of 1:30 (w/v) at 60 °C for 2 h in a temperature-controlled magnetic stirrer at a fixed stirring speed of 200 rpm for removing lipids, pigments and other small molecules. Subsequently, the residue was dried at ambient temperature and extracted thrice using reflux condensation with 0.3% Na_2CO_3 aqueous solution with a solid-liquid ratio of 1:30 at 95 °C for 4 h. The resulted solution was pooled, filtered, and centrifuged (5000 rpm, 30 min) to obtained supernatant from mixture for concentration using a rotary evaporator at 55 °C. After that, a quadruple volume of absolute ethanol was added into the concentrate for alcohol-precipitation at 4 °C for 24 h. The precipitates were harvested by centrifugation and completely dissolved into ultra-pure water (< 0.057 μS/cm) prepared by a Milli-Q ultrapure filtration system (Merck, Darmstadt, Germany) followed by deproteinization employing Sevag method [2]. The resulting products were dialyzed against distilled water for 72 h at room temperature with molecular weight cut-off of 3000 Da to eliminate inorganic salts and residual organic reagent. Finally, crude MCP was obtained after lyophilization.

1.2. Pretreatment of polysaccharide for monosaccharide composition analysis

The monosaccharide composition of polysaccharide was determined following the issued procedure with some modifications [3]. Briefly, 5 mg polysaccharide sample was hydrolyzed by 1 mL of 2 mol/L trifluoroacetic acid aqueous solution at 120 °C for 2 h. After that, the solution was dried by gentle nitrogen flow. The dried mixture was re-dissolved in 3 mL methanol for purification, then evaporated to dryness with nitrogen flow, and this process was repeated triple times. The sample was dissolved into 5 mL sterile water. After complete dissolution, 0.2 mL of 0.5 mol/L NaOH aqueous solution and 0.5 mL of 0.5 mol/L 1-phenyl-3-methyl-5-pyrazolinone (PMP) methanol solution was carefully transferred into 0.2 mL sample solution, respectively. After fully mixed by vortex, the reaction was conducted in a water bath at 70 °C for 1 h. The excess of PMP was removed by vortex extraction with 1 mL of chloroform for 3 times. After discarding the chloroform layer, deionized water was added to achieve isovolumetric process.

1.3. Methylation analysis

The MCP-3 samples were derivatized according to the following steps. Ten milligrams of sample were weighed and dissolved with 1 mL of primary water, reacted with 1 mL of 100 mg/mL carbodiimide for 2 h. At the end of the reaction, 1 mL of 2 mol/L imidazole was added, and the sample was divided equally into two parts, and 1 mL of 30 mg/mL of $NaBH_4$ and 1 mL of 30 mg/mL of $NaBD_4$ were each added, and the reaction was mixed for 3 h. At the end of the reaction, the reaction was terminated with 100 μL of glacial acetic acid and dialyzed for 48 h, and then freeze-dried. The lyophilized samples

were used for methylation treatment by adding 500 μL of DMSO and then 1 mg of NaOH and incubated for 30 min. After the end, the reaction was mixed with 50 μL of iodomethane solution for 1 h. After adding 1 mL of water and 2 mL of CH_2Cl_2, vortexed and mixed, centrifuged and discarded the aqueous phase, aspirated the lower dichloromethane phase, repeated the aqueous wash three times, and evaporated the lower layer. The evaporated sample was added with 2 mol/L trifluoroacetic acid 100 μL at 121 °C for 90 min and evaporated at 30 °C. Add 50 μL of 2 mol/L ammonia and 50 μL of 1 mol/L $NaBD_4$, and mix well for 2.5 h at room temperature. At the end of the reaction, add 20 μL acetic acid to terminate the reaction, blow dry under nitrogen, wash with 250 μL methanol, and repeat twice. After nitrogen blowing and drying, add 250 μL acetic anhydride, vortex, and mix for 2.5 h at 100 °C. Add 1 mL of water and let stand for 10 min. Add 500 μL CH_2Cl_2, vortex, and mix well, centrifuge, discard the aqueous phase and aspirate the lower dichloromethane phase, repeat the aqueous wash 3 times, and collect the lower layer for onboard detection.

The samples were filtered through an organic filter membrane of 0.22 μm and analyzed by gas chromatography–mass spectrometry system (GC-MS). GC-MS analysis conditions: the chromatographic system was sampled on an Agilent Technologies Inc. (Agilent Technologies Inc., CA, USA) with high-purity helium (purity not less than 99.999%) as the carrier gas and a flow rate of 1.0 mL/min and an inlet temperature of 260 °C. The injection volume was 1 μL, and the sample was injected in a split flow with a split ratio of 10 : 1. The solvent was delayed for 2.2 min, held at 50 °C for 1.0 min, ramped up to 130 °C at 50 °C/min, and ramped up to 230 °C at 3 °C/min, and held for 2 min.

1.4. Physicochemical determination of polysaccharide

1.4.1. Polysaccharide yield

The measurement for the yield of polysaccharide was conducted using phenol sulfuric acid method according to Huang et al. [1]. Briefly, the absorbances of varying concentrations of polysaccharide were measured at 490 nm after reaction with 6% (m/v) phenol aqueous solution and concentrated sulfuric acid. The concentrations were obtained from the following formula according to a standard curve setup by glucose standard solution.

$$Polysaccharide\ yield\ (\%) = \frac{D \times V \times C}{W} \times 100\%$$

where D is the dilution multiple of polysaccharide solution; V is the volume of polysaccharide solution (mL); C is the concentration of glucose standard solution (g/mL); W is the weight of polysaccharide (g).

1.4.2. Protein content

Protein content in polysaccharide was determined using Coomassie Brilliant Blue G250 staining method. The absorbance was detected at 595 nm after polysaccharide sample mixed with 0.2% (m/v) Coomassie Brilliant Blue G250 dye aqueous solution. The protein content in polysaccharide was calculated using a standard curve of protein standard.

1.4.3. Polyphenol content

Polyphenol content in MCP was determined by foline-phenol method according to Chinese standard refer to GB/T 8313-2018. The resulting blue compound generated from the

hydroxyl groups in polyphenol oxidized by foline-phenol reagent. The absorbance was detected at 765 nm after 60 min oxidation reaction. Gallic acid was used as correction standard curve to determine the content of polyphenol. Polyphenol content in MCP was calculated by following formula:

$$Polyphenol\ content\ (\%) = \frac{D \times V \times C}{W} \times 100\%$$

where D is the dilution multiple of MCP solution; V is the volume of MCP solution (mL); C is the concentration of polyphenol (g/mL); W is the weight of MCP (g).

1.5. In vitro antioxidant ability of MCP-3

1.5.1. 2, 2-diphenyl-1-picrylhydrzyl (DPPH) free-radical scavenging ability

The DPPH radical scavenging ability was measured using the protocol reported by Tang et al. [4], with some modifications. Two milliliters of MCP-3 in deionized water with concentration of 0.1, 0.2, 0.3, 0.4 and 0.5 mg/mL were fully mixed with 2 mL of 1 mmol/L ethanolic solution of DPPH reagent. After sharply vortexed, the mixture was left for 60 min at room temperature in the dark and measured the absorbance at the wavelength of 517 nm. As a blank, 2 mL absolute ethanol and 2 mL pure water were replaced DPPH and MCP-3 sample, respectively, to reacted at the identical conditions, which the resulting absorbances were denoted as A_{Blank1} and A_{Blank0}, respectively. Radical scavenging ability was calculated using following equation:

$$\text{DPPH radical scavenging ability (\%)} = \frac{1-(A_{Sample}-A_{Blank1})}{A_{Blank0}} \times 100\%$$

where A_{Sample}: the absorbance after adding both MCP-3 sample and DPPH; A_{Blank0}: the absorbance of the blank with absolute ethanol, pure water, and DPPH; A_{Blank1}: the absorbance of the blank with absolute ethanol, pure water, and MCP-3 sample.

1.5.2. Superoxide free-radical scavenging ability

To measure the capacity of MCP-3 to scavenge superoxide free radicals, 1 mL polysaccharide sample (0.1, 0.2, 0.3, 0.4 and 0.5 mg/mL) was firstly added into 5 mL Tris-HCl (50 mmol/L, pH=8.2) preheated in a water bath at 25 °C for 20 min. Subsequently, 0.5 mL pyrogallic acid of 3 mmol/L preheated at 25 °C was added into the mixture which was then allowed to stand for 5 min in a water bath at 25 °C. After reaction, 1 mL HCl aqueous solution (8 mmol/L) was added for reaction termination. The absorbance of resulting final product was measured at 299 nm [5]. The same volume of deionized water was substitute for sample and pyrogallol in the reaction to obtain the corresponding absorbances (A_{Blank0} and A_{Blank1}), respectively. The superoxide anion clearance rate was calculated as follow:

$$\text{Superoxide free radical scavenging ability (\%)} = \frac{1-(A_{Sample}-A_{Blank1})}{A_{Blank0}} \times 100\%$$

where A_{Sample}: the absorbance after adding both MCP-3 sample and pyrogallol; A_{Blank0}: the absorbance of the blank with deionized water and pyrogallol; A_{Blank1}: the absorbance of the blank with deionized water and MCP-3 sample.

1.5.3. Hydroxyl free-radical scavenging ability

The assay of hydroxyl free-radical scavenging ability was carried out according to the

method of Yang et al. [5] and Tang et al. [6]. Briefly, 1 mL polysaccharide solution (0.1, 0.2, 0.3, 0.4 and 0.5 mg/mL), 1 mL $FeSO_4$ aqueous solution (2 mmol/L) and 1 mL salicylate ethanol solution (6 mmol/L) were transferred and fully mix in 10 mL stopper tube. Subsequently, the tube was incubated in a water bath at 37 °C for 30 min after 1 mL H_2O_2 aqueous solution (6 mmol/L) thoroughly vortexed. The absorbance reading was immediately measured at a wavelength of 510 nm when the reaction finished. Hydroxyl radical scavenging ability was calculated as follows:

$$\text{Hydroxyl radical scavenging ability (\%)} = \frac{1-(A_2-A_1)}{A_0} \times 100\%$$

where A_0 is the absorbance of control without MCP-3 sample; A_1 is the absorbance after adding both MCP-3 sample and H_2O_2; A_2 is the absorbance value of the MCP-3 sample without H_2O_2.

1.5.4. Nitroso ion scavenging ability

The test of nitroso ion scavenging ability was determined by naphthalene hydrochloride ethylenediamine method according to Chinese standard referred by GB 5009.33-2016. In summary, 1 mL MCP-3 solution (0.1, 0.2, 0.3, 0.4 and 0.5 mg/mL) was completely mixed with 1 mL $NaNO_2$ (5 μg/mL), and then incubated in water bath at 37 °C for 30 min. Next, the mixture stood still for 5 min after mixed with 1 mL 4 mg/mL 4-aminobenzenesulfonic acid. After mixed with 0.5 mL 2 mg/mL naphthalene ethylenediamine hydrochloride and stood still for 15 min, the absorbance of final resulting mixture was measured at a wavelength of 540 nm. Nitroso ion clearance rate was calculated using followed formula:

$$\text{Nitroso ion clearance rate (\%)} = \frac{A_0-A_1}{A_0} \times 100\%$$

where A_0 is the absorbance of the control adding deionized water instead of MCP-3 sample; A_1 is the absorbance after adding MCP-3 sample.

1.5.5. 2, 2'-azinobis-(3-ethylbenzthiazoline-6-sulphonate) (ABTS) free-radical scavenging ability

The measurement of ABTS free-radical scavenging rate for MCP-3 was used to the method reported previously [6]. In brief, 7 mmol/L ABTS aqueous solution was thoroughly mixed with 2.45 mmol/L potassium persulfate aqueous solution in equal volume. The mixture was held in darkness at 4 °C for 16 h to obtain the stable absorbance at 734 nm. After standing still, the radical solution was further diluted with deionized water until the initial absorbance value of 0.7 ± 0.02 at 734 nm was reached. For analysis of MCP-3, 3.9 mL diluted ABTS solution was added into 0.1 mL polysaccharide and then shaken well. Absorbance was taken after 6 min at 734 nm. Ascorbic acid and BHT were used as positive controls.

$$\text{ABTS clearance rate (\%)} = \frac{A_0-A_1}{A_0} \times 100\%$$

where A_0 is the absorbance of the control adding deionized water instead of MCP-3 sample; A_1 is the absorbance after adding sample.

1.5.6. 2-phenyl-4, 4, 5, 5-tetramethylimidazoline-3-oxide-1-oxyl (PTIO) free-radical scavenging ability

The assay of PTIO free-radical scavenging ability was determined followed the method

reported by Li [7] and Yang [8]. Initially, 0.8 mL PTIO reagent (0.15 mg/mL) was transferred into 0.2 mL sample solution (0.1, 0.2, 0.3, 0.4 and 0.5 mg/mL), and then fully vortexed. After incubation at ambient temperature for 30 min, the absorbance of the resulting mixture was measured at 557 nm. The formula for calculating the inhibition of PTIO was as follows:

$$\text{Inhibition (\%)} = \frac{A_0 - A_1}{A_0} \times 100\%$$

where A_0 is the absorbance of the control adding deionized water instead of MCP-3 sample; A_1 is the absorbance after adding sample.

1.5.7. Ferric reducing ability (FRAP)

In vitro ferric reducing ability of MCP-3 was measured following the protocol described by Oyaizu [9]. In brief, an aliquot of 1 mL sample solution (0.1, 0.2, 0.3, 0.4 and 0.5 mg/mL) was added into 2.5 mL of sodium phosphate buffer (0.2 mol/L, pH=6.6) and 2.5 mL 1% (m/v) potassium ferricyanide aqueous solution. After thoroughly blended, the mixture was incubated in a water bath at 50 °C for 20 min, and then 2.5 mL 10% (m/v) trichloroacetic acid aqueous solution was immediately added. Subsequently, the supernatant of 2.5 mL was drawn into 2.5 mL deionized water and 1 mL 0.1% (m/v) $FeCl_3$ aqueous solution for mixture after centrifugation at 3000 rpm for 10 min. The absorbance measured at 700 nm after standing for 10 min.

Tables

Table S1. Reaction program of RT-qPCR for mRNA relative expression of pro-inflammatory cytokines.

**Procedure**	**Cycles**	**Temperature (°C)**	**Time (s)**
Preincubation	1	95	3
2 step amplification	40	95	5
		60	30
		95	60
Melting	1	55	30
		95	1
Cooling	1	37	30

Table S2. Sequences of primers used in RT-qPCR analysis for mRNA relative expression of pro-inflammatory cytokines.

Genes	Forward primer (5′-3′)	Reverse primer (5′-3′)
β-actin	CATGTACGTTGCTATCCAGGC	CTCCTTAATGTCACGCACGAT
IL-1β	ATGATGGCTTATTACAGTGGCAA	GTCGGAGATTCGTAGCTGGA
IL-6	ACTCACCTCTTCAGAACGAATTG	CCATCTTTGGAAGGTTCAGGTTG
TNF-α	CCTCTCTCTAATCAGCCCTCTG	GAGGACCTGGGAGTAGATGAG

Table S3. Physicochemical properties of each fraction of *M. chinensis* Benth polysaccharide MCP.

**Items**	**MCP**	**MCP-C**	**MCP -1**	**MCP -2**	**MCP -3**
Yield (%)	12.60	2.24	2.5	2.45	3.125
Color	brown	white	white	white	faint yellow
Polysaccharides content (%)	35.75	32.65	28.70	16.90	31.86
Protein content (%)	8.54	N. D.	N. D.	N. D.	N. D.

N. D. means not detected.

Table S4. Molecular weights of *M. chinensis* Benth polysaccharide MCP and MCP-3.

Polysaccharides	RT (min)	Mp (Da)	Mw (Da)	Mn (Da)	Peak area ratio (%)	Distribution coefficient (Mw/Mn)
MCP	39.627	11526	13700	9628	37.05	1.423
	42.654	3660	4111	3066	72.95	1.341
MCP-3	40.594	3704	16014	3273	99.59	4.8927

Table S5. Comparison of the isolation, characterization, and ameliorative effects of *M. chinensis* Benth polysaccharide against oxidative stress with the results reported in the published literatures.

***M. chinensis* Benth polysaccharide**	**Isolation**	**Characterization**		**Antioxidation**		**Reference**
	**Extraction and purification**	**Molecular weight (KDa)**	**Monosaccharide composition**	***In vitro* assay**	**Cell assay**	
Polysaccharide from fresh *M. Chinensis* Benth by hot air-dried (FMP)	Decoction and alcohol-precipitation	M_w: 44.39	Gal, GalA, Glc, Fru, Man, Rha, and Ara	DPPH: 86.78 ± 0.15% (1000 μg/mL) ABTS (IC_{50}): 280.54 μg/mL	—	[10]
Polysaccharide from fresh *M. Chinensis* Benth with storage times of 1 year (AMP)	Decoction and alcohol-precipitation	M_w: 64.34	Fru, GalA, Gal, Glc, Rha, and Ara	DPPH: 75.59 ± 0.13% ABTS (IC_{50}): 332.34 μg/mL	—	[10]
Polysaccharide from fresh *M. Chinensis* Benth (MP)	Extracted using Na_2CO_3 and alcohol-precipitation	M_w: 158	Rha, Ara, Gal, Glc, Xyl, and GalA	DPPH: 65.43% (1 μg/mL) Hydroxyl radicals: 61.79% (1 μg/mL)	—	[1]
Polysaccharide from fresh *M. Chinensis* Benth treated by dynamic high-pressure microfluidizer (DMP)	Extracted using Na_2CO_3 and alcohol-precipitation	M_w: 164	Rha, Ara, Gal, Glc, Xyl, and GalA	DPPH: 72.09% (1 μg/mL) Hydroxyl radicals: 87.14% (1 μg/mL)	—	[1]
Polysaccharide from fresh *M. Chinensis* Benth (MP)	Decoction and alcohol-precipitation	M_w: 157	GalA, Xyl, Glc, and Gal	DPPH: 75.11 ± 0.31% (1000 μg/mL)	**Raw246.7 cells induced by H_2O_2** SOD: increased to 1.12 ± 0.18 Unit (1000 μg/mL) MDA: decreased to 96.88 ± 2.52 μmol/mL (1000 μg/mL)	[11]
Polysaccharide from fresh *M. Chinensis* Benth treated with chlorosulfonic acid-pyridine method (SMP)	Decoction and alcohol-precipitation	M_w: 177	GalA, Xyl, Glc, and Gal	DPPH: 86.95% ± 0.61% (1000 μg/mL)	**Raw246.7 cells induced by H_2O_2** SOD: increased to 1.29 ± 0.20 Unit (1000 μg/mL) MDA: decreased to 67.83 ± 2.38 μmol/mL (1000 μg/mL)	[11]

M. chinensis Benth polysaccharide	**Isolation**	**Characterization**		**Antioxidation**		Reference
	**Extraction and purification**	**Molecular weight (KDa)**	**Monosaccharide composition**	***In vitro* assay**	**Cell assay**	
Polysaccharide from fresh *M. Chinensis* Benth (MP-A)	Hot-alkali extraction and alcohol- precipitation	M_w: 190	Gal, Glc, and GlcA	FRAP: 1.18 mmol/L (640 μg/mL)<br>DPPH: 59.51 ± 1.06% (320 μg/mL)<br>Hydroxyl radicals: 23.30 ± 1.09% (640 μg/mL)	**NCTC-469 cells induced by H_2O_2**<br>MDA: decreased to 0.46 U/mg protein (1 mg/mL)	[4]
Polysaccharide from fresh *M. Chinensis* Benth (MP-U)	Ultrasonic-assisted hot-alkali extraction and alcohol-precipitation	M_w: 150	Gal, Glc, and GlcA	FRAP: 0.74 mmol/L (640 μg/mL)<br>DPPH: 65.52 ± 2.76% (320 μg/mL)<br>Hydroxyl radicals: 21.40 ± 3.99% (640 μg/mL)	**NCTC-469 cells induced by H_2O_2**<br>MDA: decreased to 0.48 U/mg protein (1 mg/mL)	[4]
Polysaccharide from fresh *M. Chinensis* Benth (MP-C)	Cellulose enzyme assisted hot-alkali extraction and alcohol-precipitation	M_w: 140	Gal, Glc, GlcA, Ara, and Xyl	FRAP: 1.68 mmol/L (640 μg/mL)<br>DPPH: 88.91 ± 1.44% (320 μg/mL)<br>Hydroxyl radicals: 47.55± 2.63% (640 μg/mL)	**NCTC-469 cells induced by H_2O_2**<br>MDA: decreased to 0.43 U/mg protein (1 mg/mL)	[4]
Polysaccharide from fresh *M. Chinensis* Benth	Extracted using Na_2CO_3 and alcohol-precipitation	M_w: 1450	Gal, Glc, and Ara	DPPH: 55.59 ± 0.69% (1.69 mg/mL)<br>Superoxide radicals: 58.42 ± 1.17% (1.69 mg/mL)<br>Hydroxyl radicals: 54.36 ± 1.56% (1.69 mg/mL)	—	[12]
Polysaccharide from fresh *M. Chinensis* Benth (MCP-3)	Purification using DEAE-52 and Dextran 100 after decoction and alcohol-precipitation	M_w: 16.014	Man, Rha, GlcA, GalA, Glc, Gal, Xyl, and Ara	DPPH: 53.0 ± 6.2% (0.2 mg/mL)<br>Superoxide radicals: 48.3 ± 2.1% (0.5 mg/mL)<br>Hydroxyl radicals: 39.7 ± 1.2% (0.3 mg/mL)<br>FRAP: 69.6% ± 0.1% (0.5 mg/mL)<br>ABTS: 26.3 ± 2.9% (0.5 mg/mL)<br>PTIO: 28.3 ± 2.4% (0.2 mg/mL)<br>Nitroso ion inhibition: 16.7 ± 1.6	**Caco-2 cells induced by DON**<br>GSH-Px: increased to 0.04 U/mg protein (100 μg/mL)<br>CAT: increased to 11.55 U/mg protein (25 μg/mL)<br>SOD: increased to 88.95 U/mL (25 μg/mL)<br>MDA: decreased to 1.71 U/nmol protein (400 μg/mL)	This study

M. chinensis Benth polysaccharide	Isolation	Characterization		Antioxidation		Reference
	**Extraction and purification**	**Molecular weight (KDa)**	**Monosaccharide composition**	*In vitro* **assay**	**Cell assay**	
				(0.5mg/mL)	ROS: decreased to 1.49 (25 μg/mL)<br>IL-1β: decreased to 0.98 pg/mL (25 μg/mL)<br>IL-6: decreased to 15.55 pg/mL (25 μg/mL)<br>TNF-α: decreased to 15.02 pg/mL (25 μg/mL)<br>**NCM460 cells induced by DON**<br>GSH-Px: increased to 4.46 U/mg protein (100 μg/mL)<br>CAT: increased to 10.12 U/mg protein (400 μg/mL)<br>SOD: increased to 61.78 U/mL (100 μg/mL)<br>MDA: decreased to 0.44 U/nmol protein (100 μg/mL)<br>ROS: decreased to 1.04 (25 μg/mL)<br>IL-1β: decreased to 0.44 pg/mL (100 μg/mL)<br>IL-6: decreased to 49.97 pg/mL (25 μg/mL)<br>TNF-α: decreased to 5.65 pg/mL (25 μg/mL)	

Figure Captions

Figure S1. Microstructure images of *M. chinensis* Benth polysaccharide MCP: (A) The morphology of MCP at 100 ×, scalebar is 500 μm; (B) The morphology of MCP at 400 ×, scalebar is 100 μm.

Figure S2. Total ion chromatogram of the methylation analysis of *M. chinensis* Benth polysaccharide MCP-3.

Figure S3. Viabilities of Caco-2 and NCM460 cells evaluated by cytotoxicity assay using the MTT method. (A) Viability of Caco-2 treated by MCP-3 for 24 h; (B) Viability of Caco-2 treated by DON for 24 h; (C) Viability of NCM460 treated by MCP-3 for 24 h; (D) Viability of NCM460 treated by DON for 24 h. Note: *** $P < 0.001$.

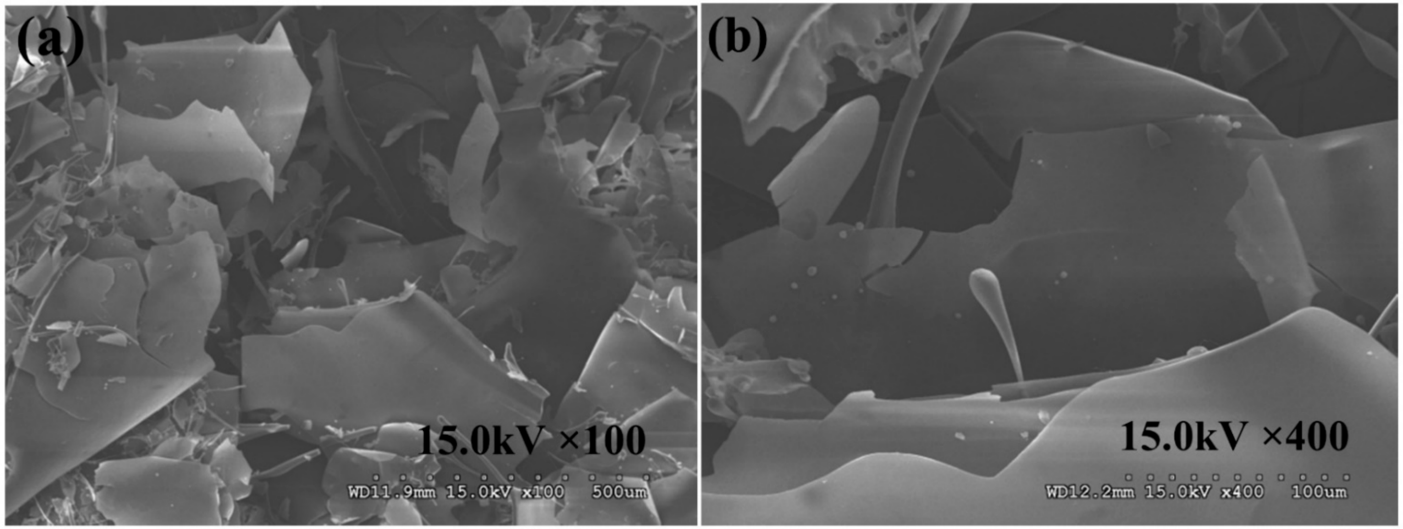

Figure S1. Microstructure images of *M. chinensis* Benth polysaccharide MCP: (A) The morphology of MCP at 100 ×, scalebar is 500 μm; (B) The morphology of MCP at 400 ×, scalebar is 100 μm.

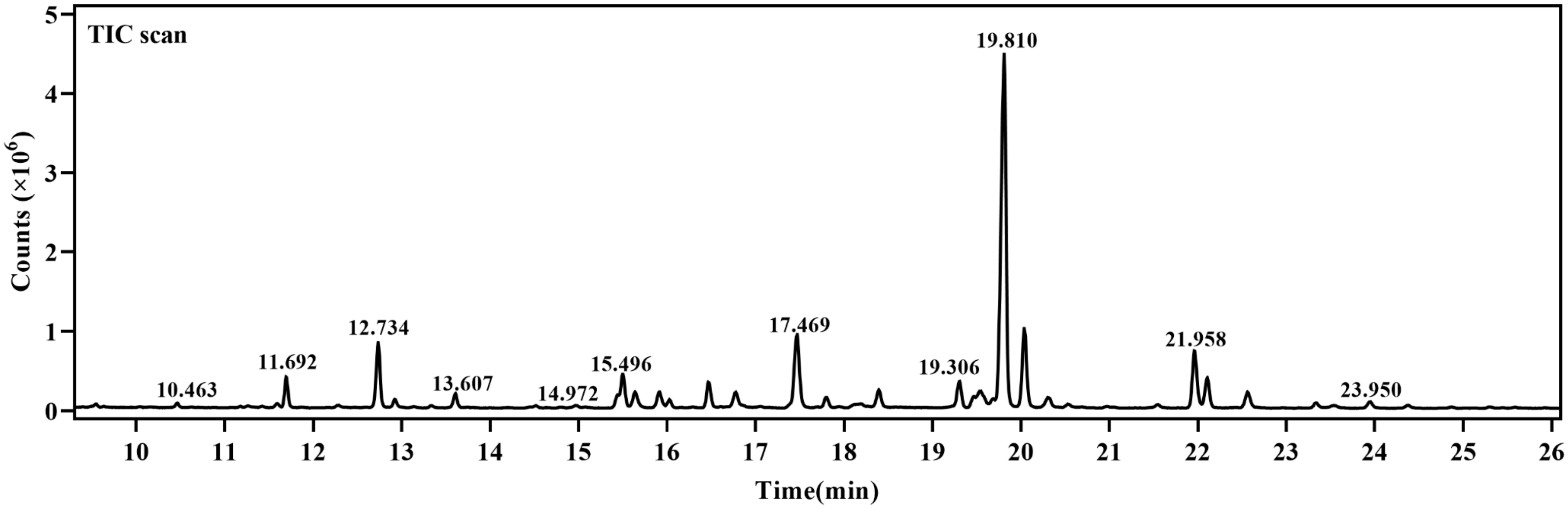

Figure S2. Total ion chromatogram of the methylation analysis for *M. chinensis* Benth polysaccharide MCP-3.

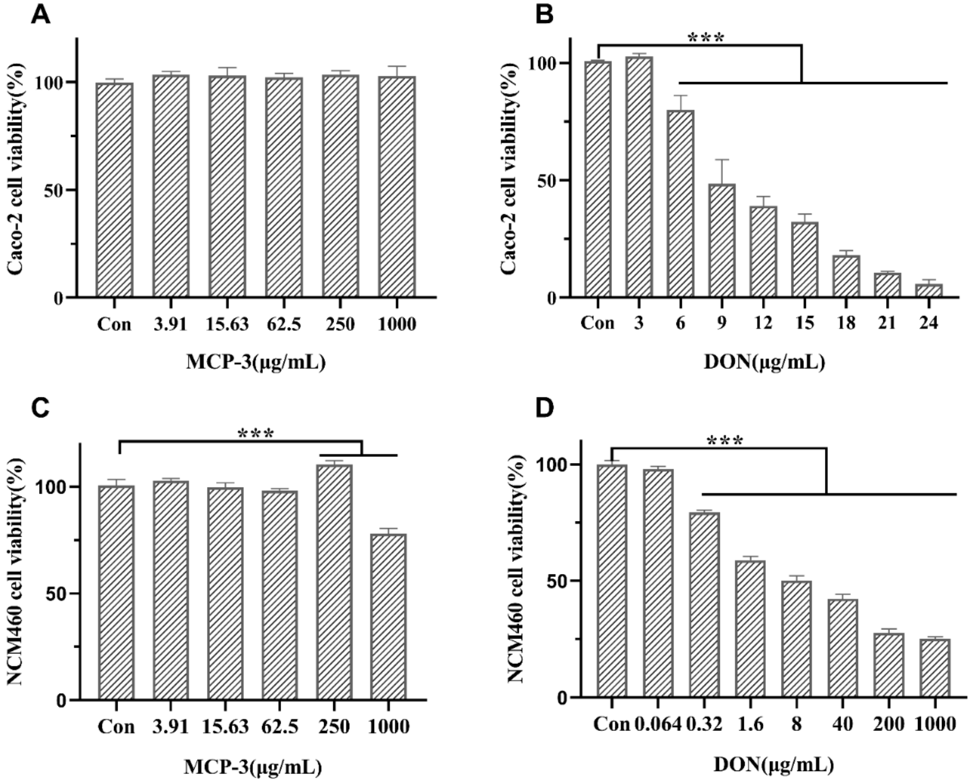

Figure S3. Viabilities of Caco-2 and NCM460 cells evaluated by cytotoxicity assay using the MTT method. (A) Viability of Caco-2 treated by MCP-3 for 24 h; (B) Viability of Caco-2 treated by DON for 24 h; (C) Viability of NCM460 treated by MCP-3 for 24 h; (D) Viability of NCM460 treated by DON for 24 h. Note: *** $P < 0.001$.

References

1. Huang, L.; Shen, M.; Zhang, X.; Jiang, L.; Song, Q.; Xie, J. Effect of high-pressure microfluidization treatment on the physicochemical properties and antioxidant activities of polysaccharide from *Mesona chinensis* Benth. *Carbohydr Polym*. **2018**, 200, 191-199. doi: 10.1016/j.carbpol.2018.07.087.
2. Pan, W.J.; Shi, L.L.; Ren, Y.R.; Yao, C.Y.; Lu, Y.M.; Chen Y. Polysaccharide ORP-1 isolated from *Oudemansiella raphanipes* ameliorates age-associated intestinal epithelial barrier dysfunction in Caco-2 cells monolayer. *Food Res Int.* **2022**, 162 (Pt A), 112038. doi: 10.1016/j.foodres.2022.112038.
3. Huo, J.; Wu, J.; Zhao, M.; Sun, W.; Sun, J.; Li, H.; Huang M. Immunomodulatory activity of a novel polysaccharide extracted from Huangshui on THP-1 cells through NO production and increased IL-6 and TNF-alpha expression. *Food Chem.* **2020**, 330, 127257. doi: 10.1016/j.foodchem.2020.127257.
4. Tang, W.; Shen, M.; Xie, J.; Liu, D.; Du, M.; Lin, L.; Gao, H.; Hamaker, B.R.; Xie, M. Physicochemical characterization, antioxidant activity of polysaccharides from *Mesona chinensis* Benth and their protective effect on injured NCTC-1469 cells induced by H_2O_2. *Carbohydr Polym.* **2017**, 175: 538-546. doi: 10.1016/j.carbpol.2017.08.018.
5. Yang, R.F.; Geng, L.L.; Lu, H.Q.; Fan, X.D. Ultrasound-synergized electrostatic field extraction of total flavonoids from *Hemerocallis citrina baroni*. *Ultrason Sonochem.* **2017**, 34, 571-579. doi: 10.1016/j.ultsonch.2016.06.037.
6. Tang, J.; Nie, J.; Li, D.; Zhu, W.; Zhang, S.; Ma, F.; Sun, Q.; Song, J.; Zheng, Y.; Chen, P. Characterization and antioxidant activities of degraded polysaccharides from *Poria cocos* sclerotium. *Carbohydr Polym.* **2014**, 105, 121-126. doi: 10.1016/j.carbpol.2014.01.049.
7. Li, X. 2-phenyl-4,4,5,5-tetramethylimidazoline-1-oxyl 3-oxide (PTIO*) radical scavenging: A new and simple antioxidant assay *in vitro*. *J Agric Food Chem.* **2017**, 65(30), 6288-6297. doi: 10.1021/acs.jafc.7b02247.
8. Yang Y.; Tan W.; Zhang, J.; Guo, Z.; Jiang, A.; Li, Q. Novel coumarin-functionalized inulin derivatives: Chemical modification and antioxidant activity assessment. *Carbohydr Res.* **2022**, 518, 108597. doi: 10.1016/j.carres.2022.108597.
9. Oyaizu, M. Studies on products of browning reaction: Antioxidative activities of products of browning reaction prepared from glucosamine. *Japanese J Nutr Diet.* **1986**, 44(6), 307-315. doi: 10.5264/eiyogakuzashi.44.307.
10. Chen, X., Xiao, W., Shen, M., Yu, Q., Chen, Y., Yang, J., Xie, J. Changes in polysaccharides structure and bioactivity during Mesona chinensis Benth storage. Curr Res Food Sci. 2022, 5, 392–400. doi: 10.1016/j.crfs.2022.01.024.
11. Huang, L.; Huang, M.; Shen, M.; Wen, P.; Wu, T.; Hong, Y.; Yu, Q.; Chen, Y.; Xie, J. Sulfated modification enhanced the antioxidant activity of Mesona chinensis Benth polysaccharide and its protective effect on cellular oxidative stress. Int J Biol Macromol. 2019, 136, 1000–1006. doi: 10.1016/j.ijbiomac.2019.06.199.
12. Lin, L.; Xie J.; Liu, S.; Shen, M.; Tang, W.; Xie, M. Polysaccharide from Mesona chinensis: Extraction optimization, physicochemical characterizations and antioxidant activities. Int J Biol Macromol. 2017, 99, 665–673. doi: 10.1016/j.ijbiomac.2017.03.040.
